# Multilayer Conductive Hybrid Nanosheets as Versatile Hybridization Matrices for Optimizing the Defect Structure, Structural Ordering, and Energy‐Functionality of Nanostructured Materials

**DOI:** 10.1002/advs.202103042

**Published:** 2021-11-10

**Authors:** Nam Hee Kwon, Xiaoyan Jin, Se‐Jun Kim, Hyungjun Kim, Seong‐Ju Hwang

**Affiliations:** ^1^ Department of Materials Science and Engineering College of Engineering Yonsei University Seoul 03722 Republic of Korea; ^2^ Department of Chemistry Korea Advanced Institute of Science and Technology (KAIST) Daehak‐ro 291 Yuseong‐gu Daejeon 34141 Republic of Korea

**Keywords:** defect structure control, enhanced charge/mass transport, hybridization, improved structural ordering, multilayer conductive nanosheet

## Abstract

The hybridization of conductive nanospecies has garnered significant research interest because of its high efficacy in improving the diverse functionalities of nanostructured materials. In this study, a novel synthetic strategy is developed to optimize the defect structure, structural ordering, and energy‐related functionality of nanostructured‐materials by employing a multilayer multicomponent two‐dimenstional (2D) graphene/metal oxide/graphene nanosheet (NS) as a versatile hybridization matrix. The hybridization of the robust trilayer, polydiallyldiammonium (PDDA)‐anchored reduced‐graphene oxide (prGO)/metal oxide/prGO NS effectively enhance the structural ordering and porosity of the hybridized MoS_2_/MnO_2_ NS through suppression of defect formation and tight stacking. In comparison with monolayer rGO/RuO_2_ NS‐based homologs, the 2D superlattice trilayer prGO/RuO_2_/prGO NS hybrids deliver better functionalities as a hydrogen evolution electrocatalyst and as a supercapacitor electrode, demonstrating the merits of hybridization with multilayer NSs. The advantages of using multilayer multicomponent conductive NSs as hybridization matrices arise from the enhancement of charge and mass transport through the layer flattening or defect suppression of the hybridized NSs and the increase in porosity, as evidenced by density functional theory calculations. Finally, the universal utility of multilayer NSs is confirmed by investigating the strong effect of the stacking order on the electrocatalytic functionality of MoS_2_/rGO/RuO_2_ films fabricated through layer‐by‐layer deposition.

## Graphical abstract

A new concept of synthetic strategy to improve various energy functionalities and mass/charge transports of nanostructured materials is developed by employing multilayer conductive graphene/inorganic/graphene nanosheet (NS) as an emerging versatile hybridization matrix. The universal usefulness of hybridization with multilayer multicomponent NSs in exploring high‐performance functional materials originates from optimization of the defect structure, structural ordering, and porosity of hybridized species.

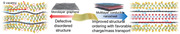

## 1 Introduction

The hybridization of nanostructured materials has received intense research attention because of its remarkable usefulness in exploring high‐performance multifunctional materials.^[^ ^1^ ^−^ ^3^ ^]^ Since the hybridization effect strongly relies on the interfacial electronic coupling between hybridized species,^[^ ^1^ ^]^ it is necessary to maximize this interfacial interaction to optimize the various functionalities of nanohybrids.^[^ ^4^ ^]^ The unusually high surface‐to‐volume ratio of conductive two dimenstional (2D) nanosheets (NSs) renders these materials highly efficient hybridization matrices via effective interfacial interactions at their wide 2D surfaces.^[^ ^5^, ^6^ ^]^ To date, a huge number of reports have been published regarding the exploration of efficient energy‐functional hybrid materials based on hybridization with conductive 2D NSs, such as reduced graphene oxide (rGO), 1T′‐MoS_2_, RuO_2_, and MXene.^[^ ^7^, ^8^, ^9^ ^]^ To further improve the energy performances of these 2D NS‐based hybrid materials, modification of the surface bonding natures of conductive NSs has been pursued as a chemical strategy to enhance the interfacial chemical interactions with hybridized species.^[^ ^10^, ^11^ ^]^ For example, hydrophilic‐surfaced RuO_2_ NS can function as an effective hybridization matrix for polar inorganic species over hydrophobic rGO NS due to a strong electrostatic dipole–dipole interaction.^[^ ^7^, ^12^ ^]^ Alternatively, the surface anchoring of polar functional groups provides another means to improve the electrochemical activities of 2D NS‐based nanohybrids via an enhancement in the interfacial interactions.^[^ ^13^, ^14^ ^]^ In contrast to these chemical approaches, the physical aspects of conductive NSs, such as their stacking structures and structural rigidities, have seldom been considered as alternative controlling factors to maximize the hybridization effect. In comparison with monolayer NSs, multilayer multicomponent conductive NSs composed of different types of interstratified monolayers would be expected to exhibit many beneficial characteristics, such as a controllable surface bonding nature, a high mechanical strength/rigidity, and enhanced transport properties. As an example, multilayer multicomponent rGO/transition metal oxide (TMO)/rGO NSs are supposed to exhibit a high structural rigidity and surface bonding controllability originating from a robust interstratified 2D structure with a tunable composition.^[^ ^15^ ^]^ Such physical features of multilayer hybrid NSs are advantageous for improving the structural ordering and charge transport properties of hybridized 2D nanospecies via the flattening of layered crystallites on a robust NS substrate. For example, an exfoliated 1T′‐MoS_2_ NS, one of the most efficient electrocatalysts for hydrogen evolution reaction (HER), suffers from low structural stability caused by the facile defect formation/lateral fracture and the resulting degradation of electrocatalyst performance,^[^ ^16^ ^]^ which might be relieved by the hybridization with robust multilayer NS substrate. Additionally, the incorporation of rigid multilayer NSs is effective in enhancing the porosities of the resulting nanohybrid due to the fact that the tight packing of flexible thin 2D NS crystallites is prevented. Such synergetic advantages of multilayer multicomponent NSs allow the circumvention of the limitation of monolayer NSs as a hybridization matrix. To date, there have been several reports about the effect of stacking layer number on the physicochemical properties and functionalities of single‐component multilayer NSs.^[^ ^17^, ^18^ ^]^ In one instance, up to the optimal stacking number, the increase of stacking numbers in the graphene oxide (GO) NSs resulted in the enhancement of specific capacitance and current density, whereas a larger GO stacking number than the optimal value caused the degrading of its electrode performance.^[^ ^17^ ^]^ In contrast to these single‐component multilayer NSs, at the time of this submission, we are unaware of any other study into the synthesis of multilayer multicomponent hybrid NSs consisting of different component monolayers or their application as building blocks for exploring highly efficient energy‐functional hybrid materials.

In this study, a novel synthetic route to high‐performance energy‐functional materials is developed by employing trilayer polydiallyldiammonium (PDDA)‐anchored rGO (denoted as prGO)/TMO/prGO hybrid NSs as a hybridization matrix. The 2D superlattice nanohybrids composed of interstratified trilayer prGO/RuO_2_/prGO NSs and MoS_2_ or MnO_2_ NSs are synthesized to verify the effectiveness of multilayer conductive NSs in exploring efficient electrocatalysts and electrode materials. To understand the higher efficiency of multilayer multicomponent NSs than monolayer single‐component NSs, evolutions of the local structural ordering and the porous nature of MoS_2_ and MnO_2_ NSs upon hybridization with trilayer prGO/TMO/prGO NS and monolayer homologs are systematically investigated by combinative spectroscopic and theoretical calculation techniques along with the effects of stacking‐pattern control on the performances of layer‐by‐layer (LbL)‐deposited MoS_2_/RuO_2_/rGO films.

## 2 Results and Discussion

### 2.1 Structural Ordering and Electronic Structure Evolution upon Hybridization with Trilayer prGO/RuO_2_/prGO NS as Compared with Monolayer Conductive NSs

**Figure 1:**
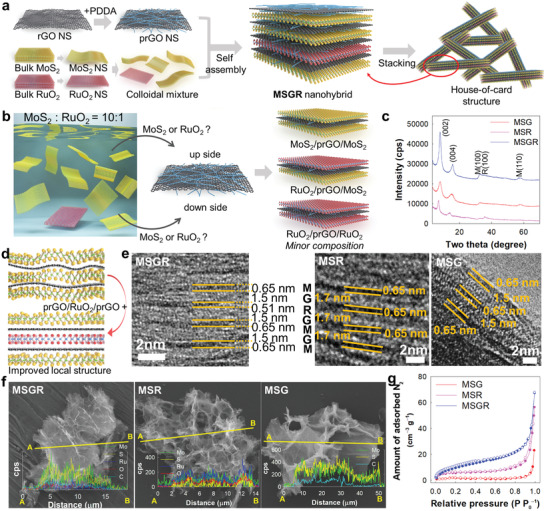
a) Synthetic scheme for superlattice MSGR nanohybrids with trilayer prGO/RuO_2_/prGO unit. b) Stacking structure models of MoS_2_/prGO/MoS_2_, RuO_2_/prGO/MoS_2_, and RuO_2_/prGO/RuO_2_ units. c) Powder XRD, d) schematic model for improved ordering structure of MSGR, e) HR‐TEM images (M: MoS_2_, R: RuO_2_, and G: prGO, respectively), f) EDS−elemental mapping data (yellow: sulfur, blue: ruthenium, green: molybdenum, red: oxygen, and cyan: carbon), and g) N_2_ adsorption−desorption isotherms of MSGR, MSG, and MSR.

As one of the main precursors for the nanohybrid, the colloidal suspension of cationic prGO NS was prepared by the reduction of modified Hummers’ method of GO and the subsequent surface anchoring of PDDA cations.^[^ ^19^ ^]^ The other precursors, namely, the colloidal monolayer RuO_2_ and the MoS_2_ NSs were synthesized via intercalation‐based exfoliation methods.^[^ ^20^, ^21^ ^]^ As shown in Figure S1a,b, Supporting Information, the highly anisotropic 2D morphologies of the monolayer prGO NSs, the RuO_2_ NSs, and the MoS_2_ NSs were evidenced by transmission electron microscopy (TEM) and atomic force microscopy (AFM), showing subnanometer‐level thicknesses of ≈0.8 nm for the MoS_2_ NSs, ≈0.7 nm for the RuO_2_ NSs, and ≈1.5 nm for the prGO NSs. In addition, zeta potential measurements clearly demonstrated the positive surface charge of the prGO NSs and the negative surface charges of the RuO_2_ and MoS_2_ NSs (Figure S1c, Supporting Information). The MoS_2_−prGO/RuO_2_/prGO nanohybrid was synthesized by an electrostatically‐driven self‐assembly process, in which the colloidal suspension of exfoliated prGO NSs was slowly added into the corresponding colloidal mixtures of RuO_2_ and MoS_2_ NSs, as depicted in **Figure** **1**a (the obtained nanohybrid is denoted as MSGR). The detail synthetic process is provided in the Experimental Section, Supporting Information. To determine the optimal composition, several MSGR nanohybrids were synthesized with various RuO_2_/MoS_2_ ratios (0−10 wt%), since the content of conductive NS has been well‐known to have significant influence on the electrocatalyst performance of nanohybrids.^[^ ^12^, ^15^ ^]^ In the compositional ranges employed, the MSGR nanohybrid with the RuO_2_/MoS_2_ ratio of 7.5 wt% showed well‐ordered hybrid structure and an optimized electrocatalyst performance (Figures S2,S3, Supporting Information). As presented in Figure S3a,b, Supporting Information, the increase of trilayer prGO/RuO_2_/prGO NS content resulted in a significant lowering of the overpotential at 10 mA cm^−2^ (i.e., 298 mV for MSGR0, 152 mV for MSGR3, 136 mV for MSGR5, and 97 mV for MSGR7.5 ) and the accompanying depression of Tafel slopes. As can be seen clearly from Figure S3c, Supporting Information, the electrochemical impedance spectroscopy (EIS) clearly demonstrated that the incorporation of trilayer NSs reduces the radius of the semicircle in the Nyquist plots measured at −0.3 V (vs the reversible hydrogen electrode, RHE), thereby confirming the lowering of charge transfer resistance (*R*_ct_). It is worth noting that a further increase in the ratio to 10 wt% yielded mixed‐phase materials due to dismantling of the superlattice structure and phase separation of the component NS phases (see Figure S4, Supporting Information). Thus, further characterizations were carried out for the MSGR nanohybrid with the optimal RuO_2_/MoS_2_ ratio of 7.5 wt%.

During self‐assembly of the component NSs, the cationic prGO NSs were electrostatically stacked with anionic RuO_2_ and MoS_2_ NSs, yielding several types of restacked NS building units of RuO_2_/prGO/RuO_2_, RuO_2_/prGO/MoS_2_, and MoS_2_/prGO/MoS_2_. These restacked NS crystallites formed mesoporous house‐of‐card‐type stacking structures, as reported previously.^[^ ^8^, ^10^ ^]^ Considering the low molar ratio of RuO_2_/MoS_2_ (8.9%) employed in this study, formation of the RuO_2_/prGO/RuO_2_ unit via the adsorption of two RuO_2_ NSs on both sides of the prGO NS was less likely compared to formation of the other RuO_2_/prGO/MoS_2_ and MoS_2_/prGO/MoS_2_ units (Figure 1b). Considering that the pentalayer prGO/RuO_2_/prGO/RuO_2_/prGO hybrid NSs can only be formed through the simultaneous adhesion of two cationic prGO NSs on both sides of the hardly formed RuO_2_/prGO/RuO_2_ unit, the present MSGR nanohybrid was considered to contain trilayer prGO/RuO_2_/prGO hybrid NSs as the main conductive building unit, with only a negligible amount of the pentalayer homolog being present.

To verify the benefits of incorporating multilayer multicomponent conductive NSs as a hybridization matrix, binary superlattice nanohybrids of MoS_2_ NSs containing monolayer rGO NSs or RuO_2_ NSs were also synthesized for comparison, because the stacking thickness of conductive NS is supposed to affect the electrocatalytic activity of hybridized MoS_2_ NS.^[^ ^22^ ^]^ For the LbL‐ordered hybridization with anionic MoS_2_ NSs, cationic RuO_2_ NSs were prepared as a precursor by the surface anchoring of polyethyleneimine cations on exfoliated RuO_2_ NSs (denoted as pRuO_2_). The formation of monolayer pRuO_2_ NSs with a positive surface charge was confirmed by AFM and zeta potential analyses (Figure S5, Supporting Information). The electrostatically driven self‐assembly between MoS_2_ and prGO or pRuO_2_ NSs yielded binary superlattice MoS_2_−prGO or MoS_2_−pRuO_2_ nanohybrids (denoted as MSG and MSR nanohybrids). As shown in Figure 1c, the LbL‐ordered hybridization between the component NSs was confirmed by powder X‐ray diffraction (XRD) analysis, wherein a series of (*00l*) Bragg reflections can be observed in the low 2*θ* region for the MSGR, MSG, and MSR nanohybrids. The observation of well‐developed intense (*00l*) reflections for the MSGR, MSG, and MSR nanohybrids provides clear evidence for the strong preferred orientation of highly anisotropic 2D MoS_2_, RuO_2_, and prGO NSs along the [001] direction and the predominant surface exposure of the in‐plane (*hk0*) facets of component MoS_2_/RuO_2_ NSs, confirming the layer‐by‐layer‐ordered stacking of these monolayer NSs. The present XRD patterns of these nanohybrids were in good agreement with the simulated pattern of superlattice inorganic NS−prGO nanohybrid.^[^ ^14^ ^]^ Notably, the MSGR nanohybrid containing trilayer NSs displayed stronger and sharper (*00l*) XRD peaks than those of both the MSG and MSR nanohybrids. This finding can be regarded as strong evidence for a more efficient improvement in the structural ordering of the MoS_2_ monolayer because of the effective layer flattening on the robust trilayer prGO/RuO_2_/prGO NS substrate (Figure 1d). In addition to the (*00l*) reflections, the MSGR and MSR nanohybrids exhibited in‐plane (*100*) and (*110*) peaks corresponding to the MoS_2_/RuO_2_ phases, thereby highlighting the maintenance of their original in‐plane structures. Similarly, the MSG nanohybrid displayed in‐plane (*100*) and (*110*) Bragg reflections of MoS_2_ phase. The interstratification of MoS_2_ NSs with trilayer prGO/RuO_2_/prGO hybrid NSs was further verified by high‐resolution TEM (HR‐TEM) imaging. As shown in Figure 1e, MSGR exhibited lamellar fringes with three different spacings of ≈1.50, ≈0.65, and ≈0.51 nm for the prGO, MoS_2_, and RuO_2_ NSs, respectively. Similarly, the HR‐TEM analyses provide strong evidence for the formation of superlattice MSG and MSR nanohybrids composed of homogeneously interstratified MoS_2_ and prGO or pRuO_2_ NSs. The homogeneous hybridization between MoS_2_ and prGO/RuO_2_/prGO, prGO, or pRuO_2_ NSs was further confirmed by energy dispersive spectrometry (EDS)−elemental mapping analysis (Figure 1f). The better role of trilayer prGO/RuO_2_/prGO NSs over monolayer NSs in enhancing the porosity of the nanohybrid was clearly evidenced by N_2_ adsorption−desorption isotherm measurements. More specifically, as plotted in Figure 1g, the MSGR nanohybrid possesses a larger surface area of 40 m^2^ g^−1^ compared to MSG (5 m^2^ g^−1^) and MSR (20 m^2^ g^−1^), although the sharper (*00l*) XRD peaks of MSGR suggest the formation of highly‐crystalline thicker primary‐stacked crystallites than the MSG and MSR crystallites (Figure 1c). While the Scherrer calculation of particle size from the shapes of (*00l*) peaks gives information about the thickness of single primary‐stacked crystallite having structural coherence, the porosity of restacked 2D NS materials is mainly governed by the degree of the tight packing between primary‐stacked crystallites rather than by the thickness of single primary‐stacked crystallite itself. Thus, the observed larger surface area of MSGR can be ascribed to the prevention of the tight packing between primary crystallites by the incorporation of thick rigid trilayer prGO/RuO_2_/prGO NS into the restacked NSs. Actually, it has been well‐documented that the incorporation of rigid inorganic NS is effective in depressing the dense packing of graphene primary crystallites, resulting in the improvement of porosity.^[^ ^15^, ^23^, ^24^ ^]^

**Figure 2:**
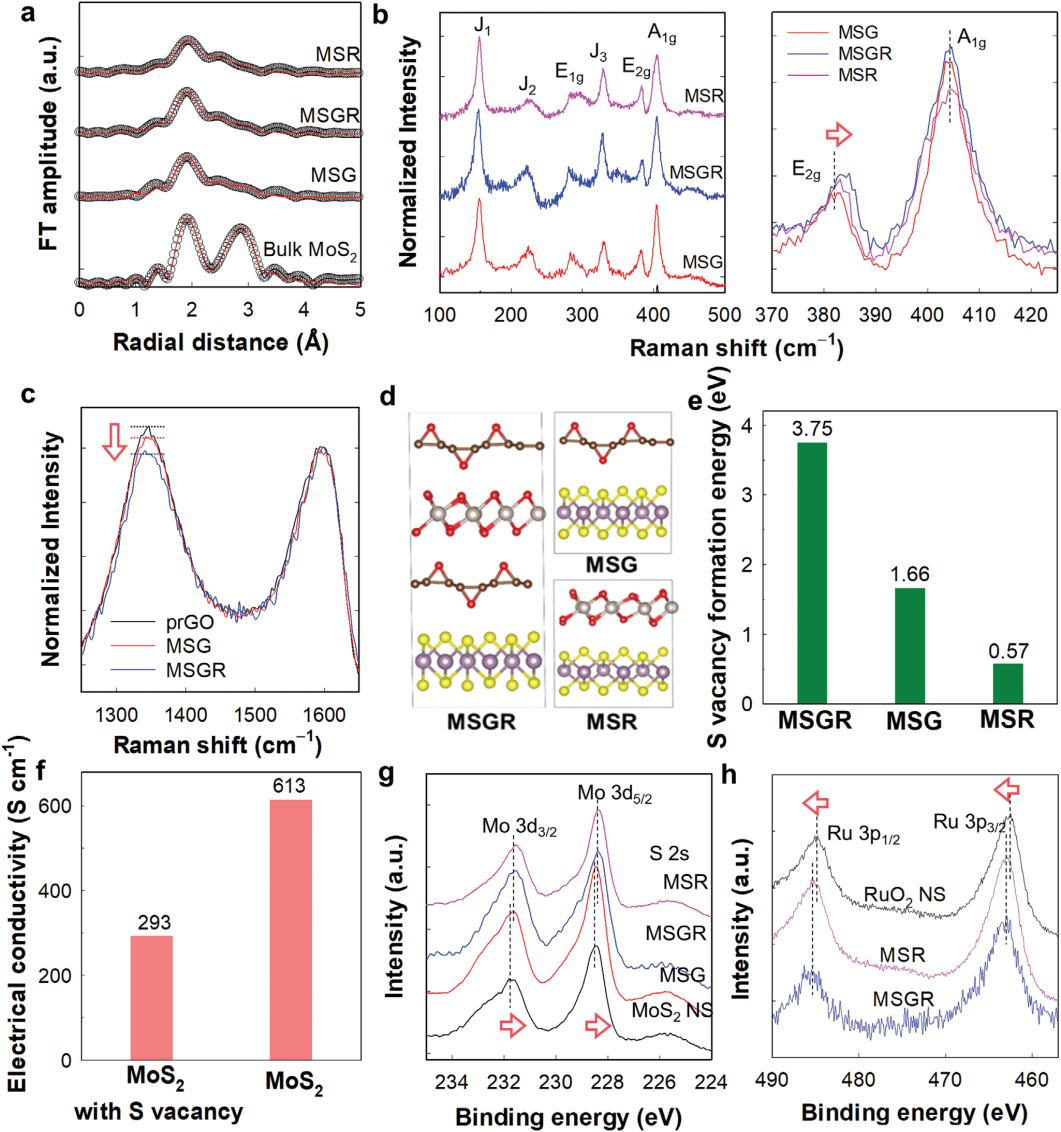
a) Mo K‐edge FT‐EXAFS spectra (circles: experimental data, red‐lines: fits), micro‐Raman spectra in b) low wavenumber and c) high wavenumber region, d) DFT model structures of MSGR, MSG, and MSR superlattice structures (Mo, S, Ru, C, and O are colored by purple, yellow, white, brown, and red, respectively), e) DFT‐calculated S vacancy formation energy of MSGR, MSG, and MSR, f) calculated electrical conductivity of vacancy‐free MoS_2_ and MoS_2_ with a S monovacancy, and g) Mo 3d XPS and h) Ru 3p XPS of MSGR, MSG, and MSR.

The local structural evolution of the MoS_2_ NSs upon hybridization with trilayer or monolayer NSs was then investigated using extended X‐ray absorption fine structure (EXAFS) analysis and micro‐Raman spectroscopy.^[^ ^25^ ^]^ As can be seen in the Mo K‐edge EXAFS spectra of **Figure** **2**a, all MSGR, MSG, and MSR nanohybrids commonly produced typical Fourier‐transformed (FT)‐EXAFS spectral features of 1Tʹ‐MoS_2_ phase, that is, three peaks at ≈1.8, ≈2.4, and ≈2.9 Å, which correspond to the (Mo−S) and two different (Mo−Mo) shells, respectively.^[^ ^26^ ^]^ This observation clearly demonstrates that stabilization of the metallic 1Tʹ‐MoS_2_ phase took place upon hybridization with the conductive NSs.

In addition, all present nanohybrids displayed notably weaker FT intensities than the bulk MoS_2_, reflecting the creation of significant structural disorder caused by the exfoliation process. A closer inspection revealed that the peak intensity was stronger for MSGR than for MSG and MSR, reflecting the higher efficiency of the trilayer prGO/RuO_2_/prGO NSs in improving the structural ordering and crystallite flattening of the hybridized 2D species than the monolayer NSs. For quantitative determination of the local structural evolution upon hybridization, non‐linear curve fitting analysis was carried out for all Mo K‐edge EXAFS spectra presented herein. As can be seen in Figure 2a, the EXAFS spectra of the nanohybrids were well reproduced with the 1Tʹ‐MoS_2_ structure, clearly demonstrating that stabilization of the metallic 1Tʹ‐MoS_2_ phase took place in these materials. The coordination numbers (CNs) of the (Mo−S) and (Mo−Mo) bonding pairs were found to be larger for the MSGR nanohybrid, which contains trilayer hybrid NSs than for MSG and MSR (i.e., based on monolayer NSs), see **Table** **1**. This provides strong evidence for improved structural ordering with depressed sulfur vacancy creation and layer flattening of the MoS_2_ crystallites upon restacking with a robust trilayer prGO/RuO_2_/prGO NS substrate. The depression of sulfur vacancy formation upon the hybridization with trilayer prGO/RuO_2_/prGO NS substrate is cross‐confirmed by the higher S/Mo ratio of MSGR than those of MSG and MSR, see Table S1, Supporting Information.^[^ ^27^ ^]^

**Table 1:** Results of non‐linear least‐squares curve fittings for the Mo K‐edge EXAFS spectra for MSGR, MSG, MSR, and bulk MoS_2_

Material	Bond pair	CN	*R* [Å]	*σ* ^2^ [10^−3^ × Å^2^]
MSGR^a)^	(Mo−S) (Mo−Mo) (Mo−Mo)	4.7 1.6 1.6	2.41 2.79 3.18	5.21 6.46 5.87
MSG^b)^	(Mo−S) (Mo−Mo) (Mo−Mo)	4.3 1.4 1.4	2.41 2.79 3.18	4.97 7.09 4.88
MSR^c)^	(Mo−S) (Mo−Mo) (Mo−Mo)	4.1 1.4 1.4	2.41 2.79 3.18	5.14 6.17 6.53
Bulk MoS_2_ ^d)^	(Mo−S) (Mo−Mo)	6.0 6.0	2.41 3.16	3.21 2.48

^^ The curve fitting analysis was performed for the range of ^a)^1.074−R−3.835 Å and 2.850−*k*−12.550 Å^−1^;

^b)^ 0.736−R−3.866 Å and 2.900−*k*−12.500 Å^−1^;

^c)^ 0.736−R−3.835 Å and 2.900−*k*−12.500 Å^−1^;

^d)^ 1.074−R−3.774 Å and 2.900−*k*−12.750 Å^−1^.

The higher efficiency of the trilayer prGO/RuO_2_/prGO NSs in enhancing the structural ordering of the hybridized 1Tʹ‐MoS_2_ component was further confirmed by micro‐Raman spectroscopy.^[^ ^28^ ^]^ As can be seen clearly from Figure 2b, the E_2g_ phonon line exhibited a higher wavenumber for MSGR than for MSG and MSR. Due to the fact that the energy of this vibration is inversely proportional to the disorder of the MoS_2_ lattice,^[^ ^29^ ^]^ this observation can be regarded as further confirmation of the improved structural ordering upon hybridization with trilayer prGO/RuO_2_/prGO NSs. In addition, in the high wavenumber region (Figure 2c), the MSGR nanohybrid displayed a lower D/G intensity ratio than MSG and prGO, clearly demonstrating that depression of the structural disorder of rGO also took place upon hybridization with the trilayer conductive NSs.^[^ ^30^ ^]^ Such enhancement in the local structural ordering with the depression of crystal vacancy formation upon hybridization with the trilayer prGO/RuO_2_/prGO NSs was further evidenced by combinative computational simulation analysis. As presented in Figure 2d,e, the density functional theory (DFT) calculations found that the superlattice formation with inorganic NS suppresses the sulfur vacancy formation. When the MoS_2_ NS is hybridized with either monolayer RuO_2_ or graphene, the sulfur monovacancy formation energy, $\Delta E_{V(S)}^{F}$ upon the superlattice formation was found to be 1.66 eV for MSG and 0.57 eV for MSR, which are much smaller than that for MSGR (3.75 eV), emphasizing the better role of trilayer prGO/RuO_2_/prGO NS in depressing the sulfur vacancy formation, as compared with monolayer prGO and pRuO_2_ NSs. Additionally, the DFT calculations clearly demonstrated that the formation of S vacancy dramatically reduces the electrical conductivity of MoS_2_. Under relaxation time (*τ*_relax_) approximation, where *τ*_relax_ is set to be 6 fs that can reproduce the experimental conductivity of the high quality 1T′‐MoS_2_,^[^ ^31^ ^]^ the introduction of S vacancy to the 1T′‐MoS_2_ reduces the electrical conductivity from 613 to 293 S cm^−1^ (Figure 2f). Based on the present DFT calculation, the suppression of S vacancy upon the hybridization with multilayer NS is supposed to improve the charge transport behavior of MoS_2_ NS, which is beneficial for improving the electrocatalyst performance of MoS_2_.

The interfacial charge transfer between MoS_2_ and the conductive NSs was also probed using surface‐sensitive X‐ray photoelectron spectroscopy (XPS). As illustrated in Figure 2g, both the RuO_2_ NS‐containing MSGR and MSR nanohybrids exhibited notably lower binding energies (BEs) for the Mo 3d XPS features than the MoS_2_ NSs, whereas nearly identical BEs appeared for rGO‐based MSG and the MoS_2_ NSs, thereby confirming the occurrence of more efficient electron injection from the prGO/RuO_2_/prGO or RuO_2_ NSs into the MoS_2_ NSs. Peak deconvolution analysis of the Mo 3d XPS data (Figure S6, Table S2, Supporting Information) clearly demonstrated an increase in the 1Tʹ‐MoS_2_ content upon hybridization with the prGO/RuO_2_/prGO NSs and the RuO_2_ NSs, which confirmed the effective stabilization of the metallic 1Tʹ‐MoS_2_ phase. Considering that an electron injection from conductive NSs to MoS_2_ NSs can stabilize the 1Tʹ‐MoS_2_ phase via the formation of a stable t_2g_^3^ configuration,^[^ ^32^ ^]^ the increase in the 1Tʹ‐MoS_2_ content in the MSGR and MSR nanohybrids can be ascribed to the enhanced interfacial electronic couplings in these materials. Indeed, the interfacial charge transfer from the RuO_2_ NSs to the MoS_2_ NSs was confirmed by Ru 3p XPS analysis. As depicted in Figure 2h, both the MSGR and MSR nanohybrids displayed slightly higher BEs for the Ru 3p peaks than the RuO_2_ NSs, confirming that an increase in the Ru oxidation state took place due to interfacial charge transfer. The interfacial charge transfer in the present nanohybrids was further supported by DFT calculation. For this calculation, the PDDA layer was excluded in the electronic structure because of its large band gap of polymer system which has less effect on band alignment. Without including the PDDA layer, electrons are transferred to the MoS_2_ layer in the calculation of the superlattice, which can be also monitored from the shift of the DFT‐calculated Mo 3d level (−222.086 eV → −219.734 eV). To confirm that such a charge transfer can occur with a larger spacing, we further constructed a simulation cell by expanding the c‐lattice parameter by twofold (Figure S7, Supporting Information). In this case, the Mo 3d core level was still calculated to be −219.472 eV, which is 2.614 eV higher than the Mo 3d core level of the pristine MoS_2_. According to several previous studies about PDDA‐based hybrid systems,^33^, ^34^, ^35^ PDDA layer was reported to act as a charge transfer mediator that can accelerate charge transfer, which asserts neglectable effect of the exclusion of PDDA layer in the electronic structure model.

### 2.2 Higher Efficacy of the Trilayer prGO/RuO_2_/prGO NSs as a Conductive Hybridization Matrix over Monolayer NS Homologs

**Figure 3:**
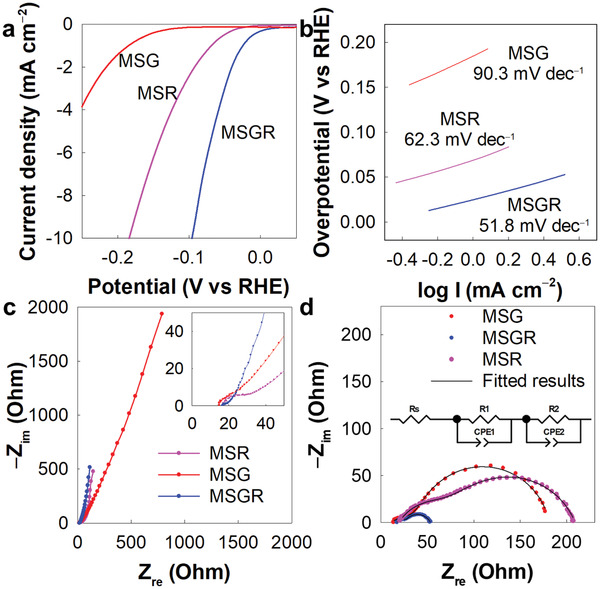
a) Linear sweep voltametry (LSV) curves, b) Tafel plots, c) Nyquist plots measured at OCV, and d) Nyquist plots measured at −0.3 V (vs RHE) for MSGR, MSG, and MSR.

To evaluate the relative efficacies resulting from the incorporation of trilayer multicomponent over monolayer single‐component conductive NSs to improve the electrocatalytic performances of hybridized MoS_2_ species, the ternary MSGR superlattice nanohybrid was tested as HER electrocatalysts for comparison with binary MSG and MSR superlattice nanohybrids. As presented in **Figure** **3**a and **Table** **2**, MSGR exhibited a significantly smaller overpotential of 97 mV at −10 mA cm^−2^ than the binary MSG (311 mV) and MSR (163 mV) species, thereby highlighting the better role of the trilayer prGO/RuO_2_/prGO NSs as a hybridization matrix. The HER activity of MSGR is comparable to the recently reported outstanding performances of MoS_2_‐based electrocatalyst in alkaline media, as presented in Table S3, Supporting Information. The crucial role of nanoscale mixing in the excellent electrocatalyst performances of the MSGR nanohybrids was confirmed by the significantly inferior HER activities of the physical mixture of MoS_2_, RuO_2_, and prGO NSs, and the precursors themselves (i.e., MoS_2_ NSs, RuO_2_ NSs, and the bulk RuO_2_) (Figure S8, Supporting Information). Of noteworthy is that the very low HER activity of RuO_2_ NS underscores its role as a conductive component rather than as an electrocatalytically‐active one. In addition to lowering the overpotential, the addition of trilayer prGO/RuO_2_/prGO NSs was also effective in improving the durability of the electrocatalyst activity, as evidenced by the chronoamperometry results presented in Figure S9, Supporting Information.

**Table 2:** HER electrocatalyst performances of MSGR, MSG, and MSR measured in 1.0 KOH electrolyte

Material	*η*_10_ [mV]	Tafel slope [mV dec^−1^]	*J*_0_ [mA cm^−2^]	*C*_dl_ [mF cm^−2^]	ECSA [cm^2^]
MSGR	97	52	0.48	24.07	42.7
MSG	311	90	0.02	10.33	18.3
MSR	184	62	0.13	5.97	10.6

The beneficial role of trilayer NSs as hybridization matrix in improving the electrocatalysis kinetics was confirmed by the smaller Tafel slope of the ternary MSGR (52 mV dec^−1^) compared to the binary MSR (62 mV dec^−1^) and MSG (90 mV dec^−1^) (see Figure 3b and Table 2). The markedly larger exchange current density (*J*_0_) value of MSGR (0.48 mA cm^−2^) compared to the corresponding values for the binary MSG (0.02 mA cm^−2^) and MSR (0.13 mA cm^−2^) offers further evidence for the remarkable influence of trilayer prGO/RuO_2_/prGO NSs in improving the HER kinetics. In addition, a steeper slope was observed in the charge current difference plot for MSGR, thereby indicating the greater double‐layer capacitance (*C*_dl_) value achieved with the trilayer prGO/RuO_2_/prGO NSs (Figure S10a, Supporting Information). As listed in Table 2, the ternary MSGR possesses an expanded electrochemical active surface area (ECSA) of 42.7 cm^2^, which is 2.3 and 4.0 times larger than the corresponding values for MSG (18.3 cm^2^) and MSR (10.6 cm^2^), respectively, thereby emphasizing the better role of the trilayer conductive NSs in increasing the electrochemical activity of the nanohybrid.

The hybridization effects of the trilayer or monolayer conductive NSs on the charge and mass transport properties were also studied using EIS analysis for binary MSG/MSR and ternary MSGR nanohybrids. In the Nyquist plots measured at the open circuit voltage (OCV) (Figure 3c), the radius of the semicircle in the high‐medium frequency region decreases in the order of MSGR < MSR < MSG. Fitting analysis for the EIS data confirmed a more prominent depression of *R*_ct_ upon the incorporation of prGO/RuO_2_/prGO NSs (8.89 Ω for MSGR, 16.1 Ω for MSR, and 45.5 Ω for MSG), thereby further demonstrating the remarkable advantage of the trilayer conductive NSs in improving the charge transfer kinetics of hybridized MoS_2_ NSs. A more efficient enhancement in the mass diffusion capability upon the incorporation of trilayer multicomponent NSs was verified by calculating the Warburg coefficient from the slope of the *Z*_re_ versus *ω* ^−0.5^ plot, which is inversely proportional to the measure of ion diffusion that takes part in the reduction−oxidation reactions across the electrode−electrolyte interface (Figure S10b, Supporting Information). More specifically, the MSGR gave a lower Warburg coefficient of 66 Ω s^−0.5^ compared to the corresponding values for MSR (84 Ω s^−0.5^) and MSG (620 Ω s^−0.5^), thereby highlighting the improved mass transport achieved when employing the trilayer prGO/RuO_2_/prGO NSs.^[^ ^36^ ^]^ The improved charge transfer kinetics achieved with the trilayer conductive NSs was cross‐confirmed by EIS measurements at −0.3 V (vs RHE), which showed a significantly smaller semicircle radius for MSGR than for MSG and MSR (Figure 3d). The observed improvements in the charge and mass transport behaviors upon hybridization with the trilayer NSs can be interpreted to result from depression of the structural disorder in the presence of fewer anion vacancies and an increase in the porosity of the MoS_2_ NSs due to layer flattening and pore formation on the robust trilayer NS substrate. This effect is mainly responsible for the better role of trilayer prGO/RuO_2_/prGO NSs in improving the HER activity of the nanohybrid.

To probe the possible interference effect of chemical composition on the HER activity of the restacked nanohybrid, another reference for a binary MoS_2_−RuO_2_ nanohybrid with the same molar ratio as MSGR was prepared by restacking a colloidal MoS_2_/RuO_2_ NS mixture with a PDDA cation (denoted as the rMSR nanohybrid), since the change of RuO_2_ NS content would cause notable change of electrocatalyst performance.^[^ ^8^, ^15^ ^]^ This system was examined due to the fact that the different charge densities of pRuO_2_ and the prGO NSs force the MSR reference to have a different ratio of MoS_2_:conductive NSs from that of MSGR to ensure that the superlattice structure is maintained. As shown in Figure S11, Supporting Information, this rMSR nanohybrid displayed a poor HER activity compared to MSGR, with a higher overpotential of 184 mV being observed at −10 mA cm^−2^. This result confirms the better role of the trilayer prGO/RuO_2_/prGO NSs as a hybridization matrix over monolayer RuO_2_ NSs. The inferior HER activity of rMSR was further confirmed by the larger Tafel slope, larger *R*_ct_ value, and smaller *C*_dl_ with lower ECSA values of this material compared to MSGR, as shown in Figure S11, Supporting Information.

### 2.3 Stacking Pattern Control in prGO/RuO_2_/prGO−MoS_2_ LbL Films

**Figure 4:**
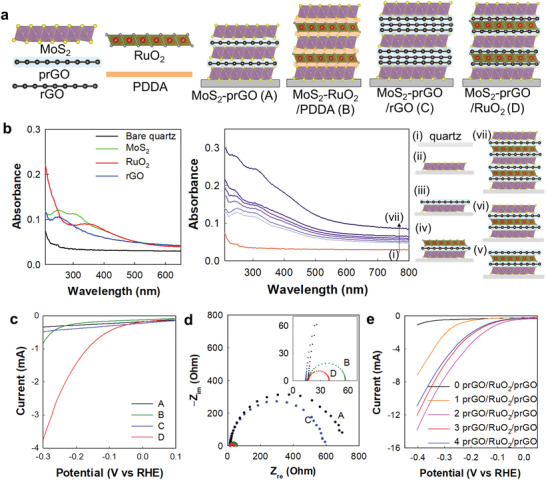
a) Stacking structures of LbL films, b) UV−vis spectra, c) LSV curves, and d) Nyquist plots measured at −0.3 V (vs RHE) for the films including prGO, RuO_2_, prGO/rGO/prGO, and prGO/RuO_2_/prGO NS as conductive NSs. e) LSV curves of LbL films consisting of five MoS_2_ layers with various prGO/RuO_2_/prGO layer.

To further verify the profound effect of the interfacial interactions of conductive NSs on the electrocatalytic activities of the hybridized electrocatalyst species, the stacking patterns of the MoS_2_, rGO, and RuO_2_ monolayers were finely controlled using the LbL deposition technique. The experimental details for the LbL deposition are provided in the Experimental Section, Supporting Information. As illustrated in **Figure** **4**a, monolayer MoS_2_ NSs were stacked with various types of conductive layers, including monolayer prGO NSs (A), monolayer PDDA‐RuO_2_ NSs (B), trilayer prGO/rGO/prGO NSs (C), and trilayer prGO/RuO_2_/prGO (D) in controlled stacking patterns. These four compositions of the LbL films were selected to compare the evolution of electrocatalyst performance upon the composition change of monolayer conductive NSs and trilayer conductive NSs. The high quality of multilayer LbL film was verified by measuring XPS data to probe the chemical bonding natures of component NSs. As presented in the full XPS survey data (Figure S12, Supporting Information), the present LbL film displayed a series of XPS signals corresponding to Mo, S, O, Ru, C, and Sn elements, confirming the successful deposition of MoS_2_, RuO_2_, and prGO NSs on the fluorine‐doped tin oxide substrate. A closer inspection for Mo 3d and Ru 3p regions revealed that the spectral features of the LbL films are nearly identical to those of MoS_2_ and RuO_2_ NSs, clarifying the maintenance of the original structure of component NSs during LbL deposition. Furthermore, the present LbL films displayed similar distinct peak shifts to those of powdery MSG, MSR, and MSGR nanohybrids, underscoring the occurrence of interfacial electron transfer between LbL‐deposited NSs.

The UV−vis spectra shown in Figure 4b clearly demonstrate the characteristic absorbance peaks of the MoS_2_ phase at ≈260 and ≈310 nm for the multilayer MoS_2_−prGO/rGO/prGO LbL film (C), wherein the intensities of these peaks become stronger upon increasing the number of MoS_2_ layers. This finding confirms the well‐controlled deposition of LbL films with an increasing number of MoS_2_ NSs. Additionally, the sequential deposition of component NS achieved by LbL technique was further evidenced by field emission‐scanning electron microscopy (FE‐SEM) and AFM analyses for the multilayer LbL film (C). As presented in Figure S13a, Supporting Information, the LbL film (C) displayed homogeneous surface morphology without any agglomerated particles, indicating the homogeneous deposition of component NSs. The AFM and surface profiler results clearly demonstrated the gradual increase of thickness and the increased coverage of substrates, confirming the consecutive deposition of component NS in each step, see Figure S13b,c, Supporting Information.

The various pattern‐controlled LbL films were then tested as HER electrocatalysts to probe the effect of the stacking order of the component NSs. As depicted in Figure 4c, a higher HER activity was observed for the RuO_2_ NS‐containing LbL films (B) and (D) than for the rGO‐based homologs (A) and (C), indicating the distinct benefit of RuO_2_ NS incorporation. Among the LbL films presented herein, the multilayer MoS_2_−prGO/RuO_2_/prGO film (D) exhibited the best HER performance, highlighting the great efficiency of the trilayer prGO/RuO_2_/prGO NSs when employed as a hybridization matrix. It is also worth mentioning that the LbL film containing the trilayer prGO/rGO/prGO unit (C) delivers an inferior HER performance to the LbL film containing trilayer prGO/RuO_2_/prGO units (D), clearly demonstrating the synergistic coupling effect between the prGO and RuO_2_ monolayers in the trilayer multicomponent NS unit. The remarkable enhancement of the electrochemical activity of MoS_2_ upon hybridization with the trilayer prGO/RuO_2_/prGO NSs was clearly evidenced from the charge current density difference plot, in which a steeper slope appears for the multilayer LbL film (D) than for the other LbL films (Figure S14, Supporting Information). This result verifies the prominently increased ECSA of the LbL film (D) (2.3 cm^2^) compared to the other LbL films, that is, 0.4, 1.5, and 0.6 cm^2^ for films (A), (B), and (C), respectively. Moreover, the beneficial contribution of the trilayer multicomponent conductive NSs to the charge transfer kinetics of the present LbL films was confirmed by EIS measurements (Figure 4d), which showed a smaller *R*_ct_ for the multilayer LbL film (D) than for the other LbL films.

The better role of the trilayer prGO/RuO_2_/prGO NSs when employed as a hybridization matrix was further demonstrated by the improved HER performance of the multilayer LbL film composed of three MoS_2_/prGO layers and one RuO_2_/prGO layer when compared to the results obtained for the film composed of RuO_2_‐free four MoS_2_/prGO layers (Figure S15, Supporting Information). This result clearly demonstrates that addition of the multilayer prGO/RuO_2_/prGO unit has an overwhelming effect on the electrocatalytic activity, which overcomes the negative effect of the accompanying decrease in the number of MoS_2_ NSs. To determine the optimal conditions for maximizing the electrocatalytic performances of the LbL films, multilayer MoS_2_−prGO/RuO_2_/prGO LbL films with several trilayer prGO/RuO_2_/prGO units were also fabricated and tested as HER electrocatalysts. As illustrated in Figure 4e, among the LbL films with five MoS_2_ layers, an increase in the trilayer prGO/RuO_2_/prGO NS number caused a gradual increase in the HER activity for up to two trilayer units. Beyond this optimal number of two, further incorporation of trilayer prGO/RuO_2_/prGO NSs did not result in any improvement in the HER performance. This result clearly demonstrates that an optimal number of trilayer prGO/RuO_2_/prGO NSs exists to improving the electrocatalytic performances of the LbL films, thereby emphasizing the importance of stacking control.

### 2.4 The Supercapacitor Electrode Performance of the Trilayer prGO/RuO_2_/prGO NS‐Based Nanohybrid

**Figure 5:**
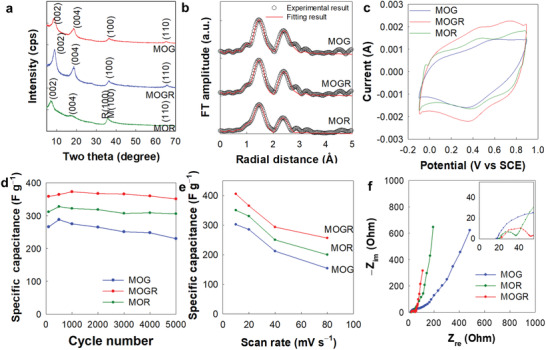
a) Powder XRD patterns, b) Mn K‐edge FT‐EXAFS, c) CV curves for the 1000th cycle, d) capacitance retention plots, e) scan rate‐dependent capacitance plots, and f) Nyquist plots for MOGR, MOG, and MOR.

The universal usefulness of multilayer multicomponent NSs as a conductive hybridization matrix to optimize the diverse energy functionalities of nanohybrids was verified by monitoring the evolution of the MnO_2_ NSs electrode performance upon hybridization with trilayer multicomponent or monolayer single‐component conductive NSs. As shown in Figure S16, Supporting Information, the TEM and AFM measurements provided clear evidence for the formation of very thin 2D monolayer MnO_2_ NS with the thickness of ≈0.6 nm. As in the case of the MSGR nanohybrids, the ternary MnO_2_−prGO/RuO_2_/prGO nanohybrid containing a RuO_2_/MnO_2_ ratio of 7.5 wt% was synthesized via an electrostatically‐driven self‐assembly process between cationic prGO/RuO_2_/prGO NSs and anionic MnO_2_ NSs (denoted as the MOGR nanohybrid). For comparison, binary homologs of superlattice MnO_2_−prGO and MnO_2_−pRuO_2_ were also prepared according to an identical synthetic process (denoted as MOG and MOR nanohybrids, respectively), because the trilayer or monolayer structure of conductive NS would have marked effect on the electrode functionality of hybridized MnO_2_ NS. As shown in **Figure** **5**a and Figure S17, Supporting Information, the formation of LbL ordered nanohybrids was confirmed by powder XRD and EDS−elemental mapping analyses. The HR‐TEM analysis for the MOGR nanohybrids provided further confirmation for the formation of well‐ordered interstratified structure composed of MnO_2_, RuO_2_, and prGO NSs, see Figure S18, Supporting Information. In addition, the FE‐SEM and HR‐TEM images (Figure S19, Supporting Information) clearly demonstrated the formation of mesoporous house‐of‐cards‐type stacking structure with the pore size of less than 50 nm and the homogeneous nanoscale mixing of the prGO/RuO_2_/prGO and MnO_2_ NSs. Similar to the case of the MSGR nanohybrids, the beneficial effect of the trilayer conductive NSs on the porosity of the restacked nanohybrid was confirmed by N_2_ adsorption−desorption isotherm measurements, which showed the greater surface area of MOGR (62 m^2^ g^−1^) compared to MOG (48 m^2^ g^−1^) and MOR (42 m^2^ g^−1^), see Figure S20a, Supporting Information. The mesoporous stacking structures of the present nanohybrids were cross‐confirmed by pore size calculation based on Barrett−Joyner−Halenda equation exhibiting the presence of mesopores, as illustrated in Figure S20b, Supporting Information.

The local structural evolution of the MnO_2_ NSs upon hybridization with trilayer multicomponent or monolayer single‐component conductive NSs was then investigated quantitatively using Mn K‐edge EXAFS analysis. As shown in Figure 5b, all nanohybrids displayed two intense FT peaks at ≈1.5 and ≈2.6 Å, which corresponded to the (Mn−O) and edge‐shared (Mn−Mn) coordination shells typical of the layered *δ*‐MnO_2_ phase.^[^ ^37^ ^]^ It is noteworthy that both FT peaks appear to be more intense for MOGR than for MOG and MOR, thereby reflecting the improvement in the structural ordering of the MnO_2_ monolayer via layer flattening and depressed defect formation on the robust trilayer prGO/RuO_2_/rGO NSs. According to the nonlinear curve fitting analysis (Table S4, Supporting Information), the CN values for the (Mn−O) and (Mn−Mn) bonds of MOGR, were significantly larger than those of MOG and MOR, highlighting the higher efficacy of the robust trilayer prGO/RuO_2_/prGO hybrid NSs over monolayer NSs in improving the structural ordering of hybridized MnO_2_ with the depression of crystal defects.

To elucidate the effect of the type of conductive NS on the electrode performance, the various MOGR, MOG, and MOR nanohybrids were tested for application as supercapacitor electrodes. In the cyclic voltammetry (CV) data shown in Figure 5c, pseudo‐rectangular shapes can be seen for the CV curves, in addition to weak redox peaks, indicating the capacitive behavior of all nanohybrids. In addition, it was found that the ternary MOGR nanohybrid displayed a larger integral area than the binary MOG and MOR homologs. Based on the CV data obtained at a scan rate of 20 mV s^−1^, the specific capacitance of MOGR was determined to be 375 F g^−1^, which is significantly higher than the corresponding values for MOG (284 F g^−1^) and MOR (268 F g^−1^), thereby verifying the beneficial effect of the trilayer prGO/RuO_2_/prGO hybrid NSs on the electrode performance of the hybridized MnO_2_ species. The specific capacitance of MOGR, which is larger than that of the corresponding physical mixture (209 F g^−1^), provided clear evidence for the importance of nanoscale mixing (Figure S21, Supporting Information). Moreover, the beneficial role of the trilayer NSs in optimizing the electrode performance of the hybridized MnO_2_ species was further confirmed by capacitance retention plots, which showed a greater specific capacitance for MOGR than for MOG and MOR (Figure 5d). As presented in the rate‐dependent capacitance plots in Figure 5e, the positive hybridization effect of the trilayer prGO/RuO_2_/prGO NSs on the specific capacitance was found to be more prominent at higher scan rates, highlighting the more effective improvement in charge transport achieved in the presence of robust trilayer conducive NSs. The beneficial hybridization effect of the trilayer prGO/RuO_2_/prGO NSs on the charge/mass transport of MnO_2_ species was then confirmed by EIS analysis. As can be seen clearly from Figure 5f, ternary MOGR exhibits a smaller semicircle radius with a lower *R*_ct_ value than the binary MOG and MOR, clearly demonstrating an improvement in the charge transport properties upon hybridization with the trilayer prGO/RuO_2_/prGO NSs. Moreover, from the slope of the Warburg plots (Figure S22, Supporting Information), a smaller Warburg coefficient of 17 Ω s^−0.5^ was determined for MOGR than for MOR (34 Ω s^−0.5^) and MOG (91 Ω s^−0.5^), confirming that mass transfer was promoted using the trilayer NSs. This significant improvement in the charge/mass transfer kinetics upon hybridization with the trilayer hybrid NSs was considered to account for the optimized electrode activity of the ternary MOGR nanohybrid.

### 2.5 Origins of the Excellent Role of the Trilayer prGO/RuO_2_/prGO NSs as a Hybridization Matrix

**Figure 6:**
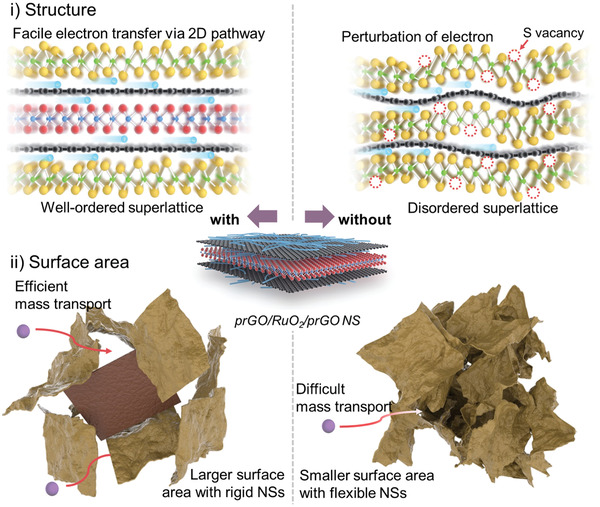
Diverse roles of trilayer prGO/RuO_2_/prGO hybrid NS in improving the electrocatalyst and electrode performances of nanohybrid in the aspect of i) structure and ii) surface area.

As illustrated in **Figure** **6**, there are several contributing factors to the excellent function of multicomponent trilayer prGO/RuO_2_/prGO hybrid NSs when employed as a conductive hybridization matrix over single‐component monolayer NS.

For example, the EIS measurements clearly demonstrate that hybridization with conductive trilayer prGO/RuO_2_/prGO NSs improves the charge/mass transfer kinetics. According to the XRD, EXAFS, and micro‐Raman analyses, an intimate interfacial binding with robust trilayer prGO/RuO_2_/prGO NSs improves the structural ordering of hybridized NSs via flattening of its layer crystallites. The elastic deformation of MoS_2_ NS creating local distortion and crystal vacancies can be effectively relieved by anchoring on rigid trilayer prGO/RuO_2_/prGO NS.^[^ ^38^ ^]^ The well‐ordered crystal structure of the hybridized 2D NSs leads to an effective improvement in the charge transport kinetics due to the suppressed perturbation of electron conduction by local structural distortion. Additionally, the intervention of thick and rigid trilayer NSs is effective in increasing the porosity of the resulting nanohybrids, thereby leading to an improvement in the mass transport behavior. The resulting charge/mass transport enhancement significantly contributes to the beneficial hybridization effect of the trilayer conductive NSs on the electrocatalyst and electrode performances of the MSGR and MOGR nanohybrids.

It should also be noted here that defect engineering is one of the most widely used chemical approaches to optimize catalytic performances,^[^ ^27^, ^39^ ^]^ wherein a large concentration of crystal defects with severe structural disordering results in disruption of the catalytic activity.^[^ ^40^ ^]^ In fact, the introduction of additional sulfur vacancies into the present 1T′‐MoS_2_ NS was found to degrade its electrocatalytic activity, see Figure S23, Supporting Information. Thus, optimization of the sulfur vacancy content plays an important role in optimizing the HER performances of MoS_2_ materials via an improvement in their surface electronic structures and electrical transport properties.^[^ ^40^ ^]^ Furthermore, the results of DFT calculations showed the detrimental effect of S vacancies on the electrical conductivity of 1T′‐MoS_2_ (Figure 2f). Thus, overall, improvements in the structural ordering and optimization of the defect contents upon hybridization with multicomponent multilayer prGO/RuO_2_/prGO NSs will be expected to provide a new chemical strategy to design and synthesize high‐performance electrocatalysts via the improvement in structural ordering. In addition, an increase in the surface area upon hybridization with the trilayer prGO/RuO_2_/prGO hybrid NSs was found to cause an enlargement in the ECSA due to the depressed tight packing of the MoS_2_/MnO_2_ NSs. The resulting increases in the ECSA and number of surface active sites also contribute to an improvement in the HER activity and the specific capacitance of the trilayer NS‐based hybrid materials. Furthermore, hybridization with the trilayer prGO/RuO_2_/prGO NSs leads to stabilization of the metallic 1Tʹ‐MoS_2_ phase. Since the 1Tʹ‐MoS_2_ phase, which contains numerous active sites in its basal plane and edge, has a higher electrocatalytic activity than the 2H‐MoS_2_ phase,^[^ ^41^ ^]^ the increased content of the 1Tʹ‐MoS_2_ phase upon hybridization can also be considered responsible for improving the electrocatalytic performances of trilayer prGO/RuO_2_/prGO NS‐based hybrid materials. Finally, in the case of the MSGR nanohybrids, the hybridization with multilayer prGO/RuO_2_/prGO NSs increases the electron density of the hybridized MoS_2_ NSs, which promotes the chemisorption of protons on the MoS_2_ surface, as depicted in Figure 6. Since the first Volmer step in the HER mechanism (H_2_O + e^−^ + M (active site) → H_ads_−M + OH^−^), which involves water dissociation and proton adsorption, is the most energetically unfavorable process,^[^ ^42^ ^]^ the increased electron density of MoS_2_ in the MSGR nanohybrids facilitates this step, which partly contributes to their improved HER performances.

## 3 Conclusion

A new versatile synthetic strategy to optimize various energy functionalities and mass/charge transports of nanostructured materials was developed by employing multilayer multicomponent conductive graphene/inorganic/graphene NSs as an emerging versatile hybridization matrix via the enhancement of structural ordering and porosity. More specifically, employing multilayer multicomponent conductive PDDA‐anchored rGO (prGO)/metal oxide/prGO (i.e., prGO/TMO/prGO) hybrid NSs as a new type of hybridization matrix was found to provide a novel and efficient methodology to explore high‐performance energy‐functional materials. The prepared MoS_2_−prGO/RuO_2_/prGO nanohybrid (denoted as MSGR nanohybrid) and the corresponding ternary MnO_2_−prGO/RuO_2_/prGO nanohybrid (denoted as MOGR nanohybrid) were found to deliver significantly better electrocatalytic and electrode functionalities than their corresponding binary homologs based on single‐component monolayer pRuO_2_ and prGO NSs. These observations demonstrate the versatility of hybridization with multilayer conductive NSs in terms of optimizing the energy functionalities of nanostructured materials. The strong interfacial interaction with the robust trilayer prGO/RuO_2_/prGO NSs was also found to be effective in enhancing the structural ordering of hybridized 2D NSs through layer flattening and depressed defect formation, which in turn led to an enhancement in the charge transport properties. In addition, the incorporation of a rigid trilayer hybrid NS into the restacked NS assembly endowed considerable porosity by preventing the tight packing of 2D crystallites; this is beneficial in promoting the mass diffusion kinetics. The resulting improvements in the charge/mass transport properties therefore accounted for the remarkable enhancements in the electrocatalytic and electrode performances upon hybridization with the trilayer conductive NSs. Considering the fact that the charge transfer kinetics and ion/mass transport behavior can play a crucial role in optimizing many other functionalities of inorganic solids such as adsorbents, ion conductors, sensors, and separator membranes,^[^ ^43^, ^44^ ^]^ the present hybridization strategy based on multilayer hybrid conductive NSs is expected to provide valuable opportunities to achieve a wide spectrum of novel functionalities. It is worth noting that the in situ formation of the multilayer hybrid NSs in the present synthetic method provides higher flexibility to design and synthesize high‐performance functional hybrid materials. Considering the vast pool of inorganic NSs, many types of multilayer multicomponent prGO/inorganic/prGO hybrid NSs could potentially be developed as new hybridization matrices for coupling with various inorganic solids. Moreover, due to the fact that the structural ordering and pore structures of the resulting nanohybrids can be further enhanced by controlling the compositions and structures of the multilayer hybrid NSs, the present synthetic strategy enables the exploitation of novel multifunctional hybrid materials. Currently, our group is working on application of the present hybridization strategy to optimize various energy‐related functionalities of inorganic nanospecies as photocatalysts for CO_2_ reduction and H_2_ production, and as electrodes for Li‐/Na‐/Mg‐ion batteries and supercapacitors; the results will be presented in due course.

## Conflict of Interest

The authors declare no conflict of interest.

## Supporting information

Supporting Information

## Data Availability

Research data are not shared.
