# Supplementary material for: Multilayer Conductive Hybrid Nanosheets as Versatile Hybridization Matrices for Optimizing the Defect Structure, Structural Ordering, and Energy‐Functionality of Nanostructured Materials
Source: Adv Sci (Weinh). 2021 Nov 10;9(2):2103042. doi: 10.1002/advs.202103042 (PMC8805630; doi:10.1002/advs.202103042)
Supplement: Supplementary file 1 — Supporting Information [file ADVS-9-2103042-s001.pdf]

© 2021 Wiley-VCH GmbH

ADVANCED SCIENCE Open Access

# Supporting Information

for *Adv. Sci.*, DOI: 10.1002/advs.202103042

Multilayer Conductive Hybrid Nanosheets as Versatile Hybridization Matrices for Optimizing the Defect Structure, Structural Ordering, and Energy-Functionality of Nanostructured Materials

*Nam Hee Kwon, Xiaoyan Jin, Se-Jun Kim, Hyungjun Kim,* and Seong-Ju Hwang**

# Supporting Information

**Multilayer Conductive Hybrid Nanosheets as Versatile Hybridization Matrices for Optimizing the Defect Structure, Structural Ordering, and Energy-Functionality of Nanostructured Materials**

*Nam Hee Kwon, Xiaoyan Jin, Se-Jun Kim, Hyungjun Kim,* and Seong-Ju Hwang**

## Experimental Section

*Synthesis*: The colloidal suspension of exfoliated two-dimenstional (2D) $MoS_2$ nanosheet (NS) was prepared by the lithiation–hydroxylation reaction of bulk $MoS_2$, as reported previously.[1] The colloidal suspension of exfoliated $RuO_2$ NS was obtained by the intercalation of tetrabutylammonium (TBA) ion into the protonated $Na_{0.2}RuO_2$.[2] For the synthesis of positively-charged polydiallyldiammonium (PDDA)-anchored reduced-graphene oxide (prGO) and polyethyleneimine (PEI) anchored $RuO_2$ ($pRuO_2$) NSs, the exfoliated graphene oxide (GO) and $RuO_2$ NSs were anchored with PDDA and PEI polycations, respectively.[3,4] The ternary superlattice **MSGR** nanohybrid was synthesized by an electrostatically-driven self-assembly of negatively-charged $MoS_2$/$RuO_2$ NSs and positively-charged prGO NS. The ratio of colloidal $MoS_2$/$RuO_2$ NS mixture and prGO NS was fixed to 5:1 in weight ratio with the $RuO_2$/$MoS_2$ ratio of 7.5wt%. The resulting **MSGR** nanohybrids were washed with distilled water and freeze-dried. For comparison, the binary superlattice **MSG** and **MSR** nanohybrids were prepared by employing prGO and $pRuO_2$ NS as hybridization matrices with the molar ratio of $MoS_2$:conductive NS (1:2.96 for **MSG**) and (1:1.16 for **MSR**). Somewhat different molar ratios of $MoS_2$:conductive NSs were applied for **MSGR**/**MSG** and **MSR** nanohybrids to construct the layer-by-layer-ordered superlattice due to their different charge densities. For comparison, the reference **rMSR** was synthesized using $MoS_2$ and $RuO_2$ NSs in the same molar ratio of 1:2.96 with the assistance of PDDA polycations as linkers. Additionally, the **MOGR** nanohybrids were synthesized by the same preparation procedure as **MSGR** nanohybrids except for the use of $MnO_2$ NS. The precursor $MnO_2$ NS was prepared by one-pot solution-based reaction with $Mn^{2+}$ precursor.[5] The ratio between $MnO_2$ and prGO NS in **MOGR** was adjusted to 2.8/1(w/w) with the $RuO_2$/$MnO_2$ ratio of 7.5wt%.

*Characterization:* Powder X-ray diffraction (XRD) patterns of the present nanohybrids were measured with X-ray diffractometer (Rigaku, $\lambda$ = 1.5418 Å, 25 °C) to study the effect of

hybridization on their crystal structures. The crystal shapes and hybrid structures of the present materials were probed with high-resolution transmission electron microscopy (HR-TEM) (Jeol JEM-2100F microscope with an accelerating voltage of 200 kV) and field-emission scanning electron microscopy (FE-SEM) (Jeol JSM-6700F microscope). The elemental distributions of the present materials were probed with energy dispersive spectrometry (EDS)–elemental mapping analysis. The evolution of porosity upon the hybridization was studied by $N_2$ adsorption–desorption isotherm analysis at 77 K using Micromeritics ASAP 2020. Mo K-edge, Mn K-edge, and Ru K-edge X-ray absorption spectra (XAS) were measured to probe the oxidation state and local symmetry of the present materials. All the present XAS data were obtained at the beam lines 8C and 10C of Pohang Accelerator Laboratory (PAL, Pohang) in Korea. The XAS measurements were carried out at room temperature using gas-ionization detectors. The XAS analysis was done according to the standard procedure, as reported previously.[6] The chemical bonding characters of the present nanohybrids were investigated with X-ray photoelectron spectroscopy (XPS) measurement (Thermo VG, UK). All the XPS data were energy-referenced to the adventitious Au 4f peak (BE = 84 eV). Micro-Raman spectroscopic analysis was carried out for the present materials using Horiba Jobin-Yvon LabRam Aramis spectrometer, in which $Ar^+$ ion laser with a wavelength of 514 nm was used as the excitation source.

*Computational Details:* Spin-polarized density functional theory (DFT) calculations were performed using Vienna Ab-initio Simulation Package (VASP) code.[7] The exchange-correlation energy was described using Perdew-Burke-Ernzerhof(PBE) functional[8] and the van der Waals interaction was corrected using zero-damping Grimme-D3 method.[9] The reciprocal space was sampled using Monkhorst-Pack[10] grid of $4 \times 2 \times 1$, and core electrons were treated by projector augmented wave (PAW) method.[11] The sulfur monovacant $MoS_2$

was modeled using 4 × 4 supercell of 1T′-$MoS_2$, where one S atom was removed to evaluate the S vacancy formation energy. The chemical potential of S was calculated using $S_8$ molecule.

*Electrocatalytic Activity Measurement:* For the test of hydrogen evolution reaction (HER) activity, the linear sweep voltametry (LSV) curves of the present materials were measured using an electrochemical working station with a three-electrode system, where Pt wire and saturated calomel electrode (SCE) (sat. KCl) were used as a counter and a reference electrode, respectively. To fabricate the working electrode, the electrode ink was prepared by dispersing the active material (2.5 mg) in the mixture of Milli-Q water, isopropyl alcohol, and 5wt% Nafion solution under sonication. 10 μL of the obtained ink was deposited on glassy carbon and dried in oven. 1 M aqueous KOH solution was used as electrolyte after the purging of $N_2$ gas for 30 min. The LSV data were collected in the potential region from −0.95 to −1.35 V (vs. SCE) at a scan rate of 5 mV $sec^{-1}$. During the electrochemical measurement, the working electrode was rotated at 1600 rpm to remove $H_2$ bubbles attached on the electrode surface. The electrochemical impedance spectroscopy (EIS) data of the present materials were measured at several potentials with a frequency range of 0.1–10000 Hz. The scan-rate-dependent charging current density was monitored by measuring cyclic voltametry (CV) curves at open circuit voltage (OCV). The electrochemical active surface areas (ECSAs) of the present materials were calculated from double-layer capacitance ($C_{dl}$) divided by specific capacitance ($C_s$). The $C_{dl}$ values were determined from the slope of charging current vs. scan rate measured by CV under the assumption that $C_s$ for flat surface in alkaline media is 40 μF $cm^{-2}$.[12]

*Layer-by-layer (LbL) Film Preparation:* The LbL hybrid films composed of $MoS_2$, $RuO_2$, prGO, and rGO NSs were deposited on the substrate of flurorine-doped tin oxide (FTO) and quartz glass.[13] Prior to film deposition, FTO glass was cleaned via the sonication under ethanol/acetone/distilled water and quartz glass was washed according to the previously-

reported process.[13] For the first step of the LbL deposition, the FTO/qaurtz glass was pre-coated by immersing it in an aqueous solution of PEI (2.5 mg $mL^{-1}$) at pH 9 for 30 min with the surface area of 1.5 ×1.5 $cm^2$, resulting in the positively-charged surface state. After being rinsed gently with distilled water, the PEI-coated substrate was dipped into dialized suspension of $MoS_2$ NS (0.08 mg $mL^{-1}$) for 20 min, followed by washing with distilled water. Next, FTO glass coated with negatively-charged $MoS_2$ NS was consecutively dipped into 20 ml of prGO NS suspension (0.08 mg $mL^{-1}$) for 20 min, followed by washing with distilled water. After coating positvely-charged prGO NS, negatively-charged $MoS_2$ or $RuO_2$ NSs were sequentially deposited with the same procedure. In each step, 20 mL of fresh colloidal suspension of dialyzed NSs (0.08 mg $mL^{-1}$) was provided, followed by washing with fresh distilled water. For the coating of negatively-charged $RuO_2$ NS onto negatively-charged $MoS_2$ NS, $MoS_2$-coated FTO was dipped into the PDDA solution (20 mg $mL^{-1}$) at pH 9 for 20 min to modify the surface charge to positive state. After the completion of successive coating process, the obtained multilayer film was dried at 40 °C.

*Supercapacitor Performance Measurement:* The supercapacitor electrode functionalities of **MOGR**, **MOG**, and **MOR** nanohybrids were studied by measuring CV in the potential range of −0.1 ~ +0.9 V (vs. SCE). All the electrochemical data were measured with a conventional three-electrode system using potentiostat (WonA Tech). The working electrode was fabricated using homogeneous slurry of the active material, acetylene black, and poly(vinylidene fluoride) in a mass ratio of 80:10:10 with *N*-methyl-2-pyrrolidone. The resulting slurry was pressed on a stainless steel substrate with the surface are of 1×1 $cm^2$ using doctor blade to generate working electrode. The SCE and Pt mesh were used as reference and counter electrodes, respectively, while 0.2 M aqueous $Na_2SO_4$ solution was employed as electrolyte.

*Statistical Analysis* : All the electrochemical measurements of electrocatalyst/electrode were repeated at least 5 times reproducing the same performance.

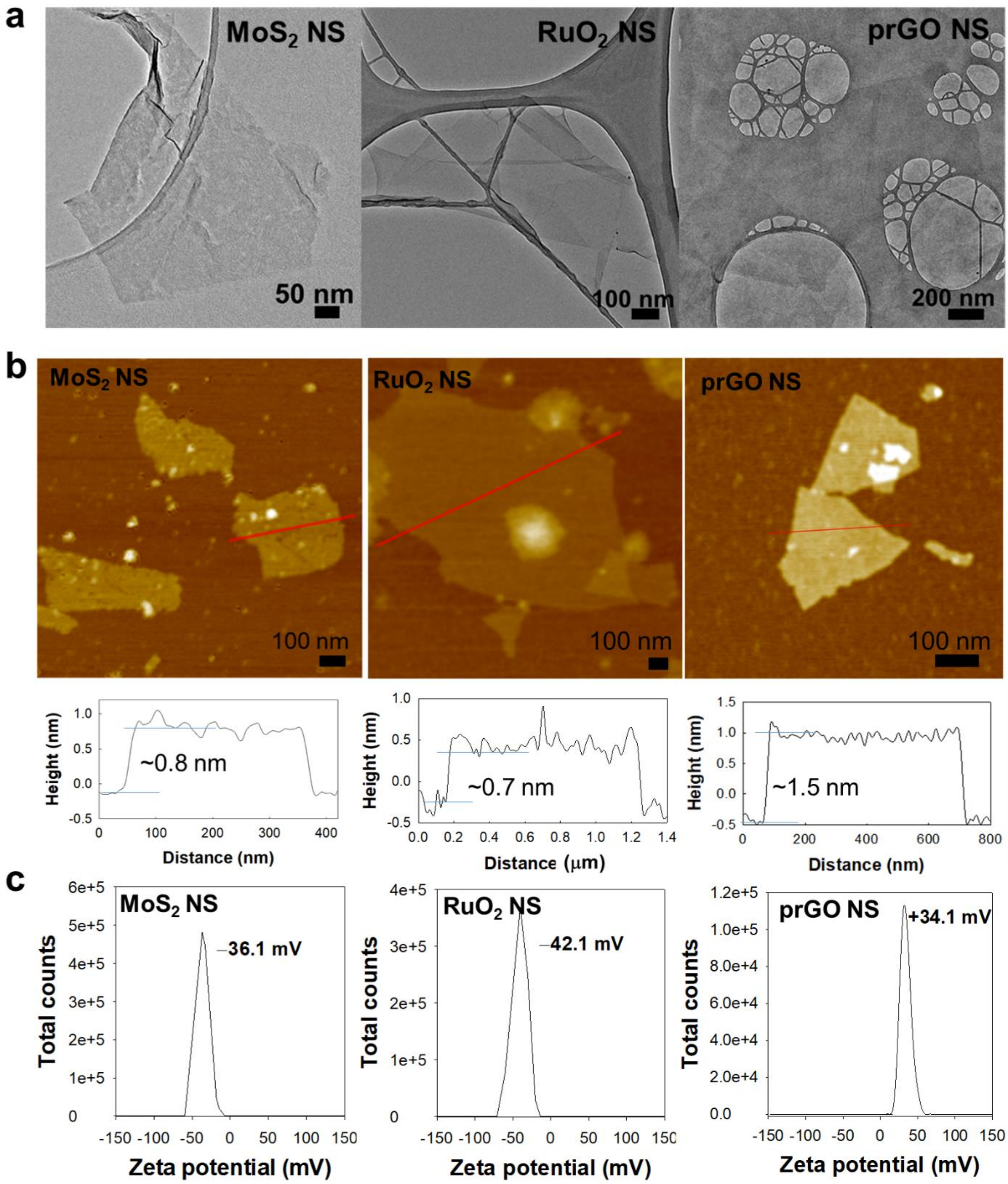


**Figure S1.** (a) TEM images, (b) AFM images, and (c) zeta potentials of $MoS_2$, $RuO_2$, and prGO NSs.

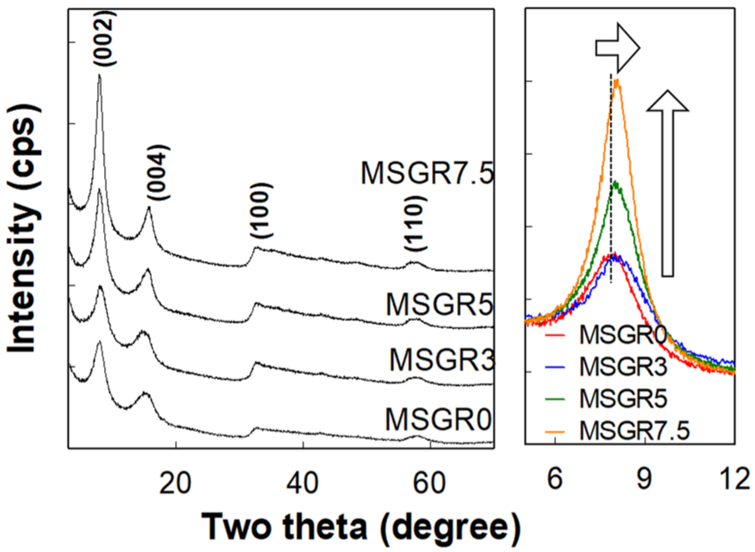


**Figure S2.** Powder XRD patterns of **MSGR** nanohybrids with several $RuO_2/MoS_2$ ratios (0, 3, 5, and 7.5wt%).

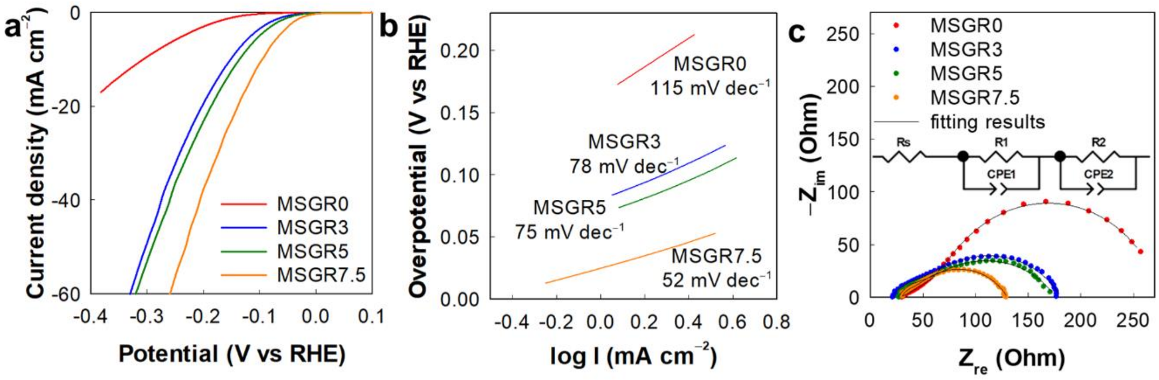


**Figure S3.** (a) LSV curves, (b) Tafel plots, and (c) Nyquist plots measured at –0.3 V (vs. RHE) for **MSGR** nanohybrids with several $RuO_2/MoS_2$ ratios (0, 3, 5, and 7.5wt%). All the electrochemical performances were measured in an alkaline electrolyte of 1.0 M KOH solution.

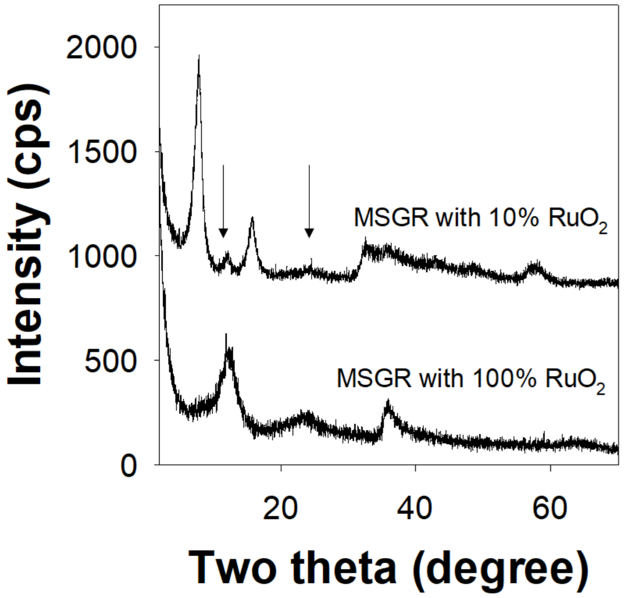


**Figure S4.** Powder XRD patterns of **MSGR** nanohybrids containing 10% and 100% $RuO_2$ NS.

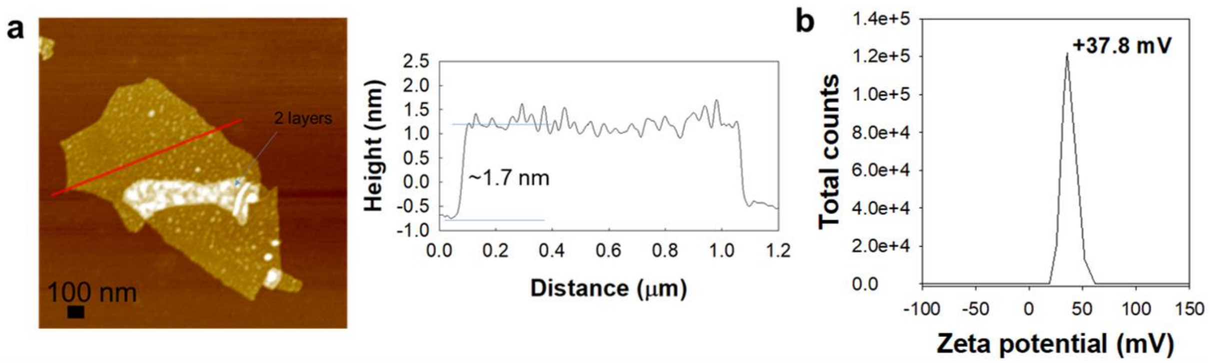


**Figure S5.** (a) AFM image and (b) zeta potential of $pRuO_2$ NS.

**Table S1.** Atomic S/Mo ratios determined by Mo 3p and the S 2p XPS for bulk $MoS_2$, **MSG**, **MSR**, and **MSGR** nanohybrids.

| Sample | S/Mo Ratio | Corrected S/Mo Ratio |
|---|---|---|
| **Bulk $MoS_2$** | 2.48 | 2.00 |
| **MSG** | 2.19 | 1.76 |
| **MSR** | 2.22 | 1.79 |
| **MSGR** | 2.24 | 1.81 |

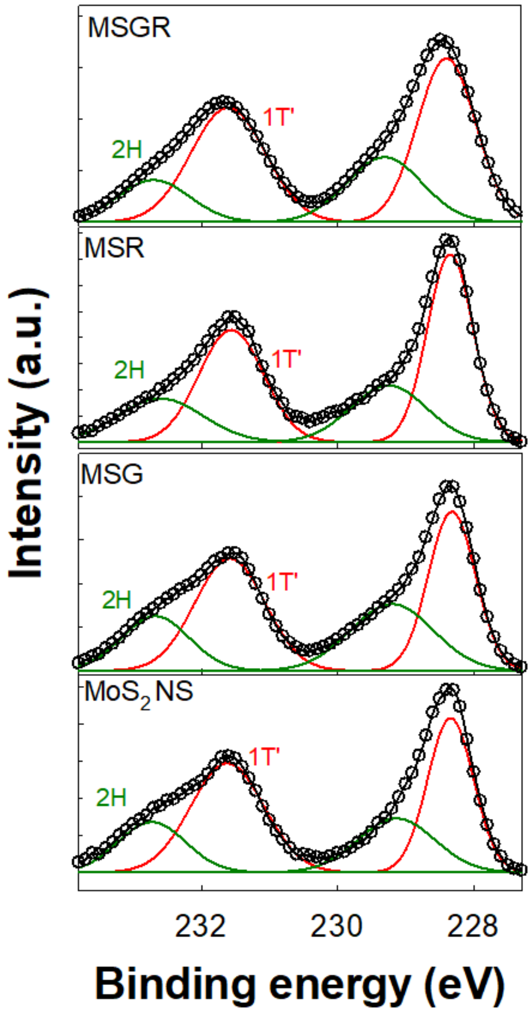


**Figure S6.** Peak deconvolution results for Mo 3d XPS of $MoS_2$ NS, **MSG**, **MSR**, and **MSGR** nanohybrids.

**Table S2.** Contents of 1T′ and 2H $MoS_2$ phases determined by Mo 3d XPS for $MoS_2$ NS, **MSG**, **MSR**, and **MSGR** nanohybrids.

| Sample | 1T′ $MoS_2$ | 2H $MoS_2$ |
|---|---|---|
| **$MoS_2$ NS** | 59% | 41% |
| **MSG** | 63 % | 37 % |
| **MSR** | 67 % | 33 % |
| **MSGR** | 70 % | 30 % |

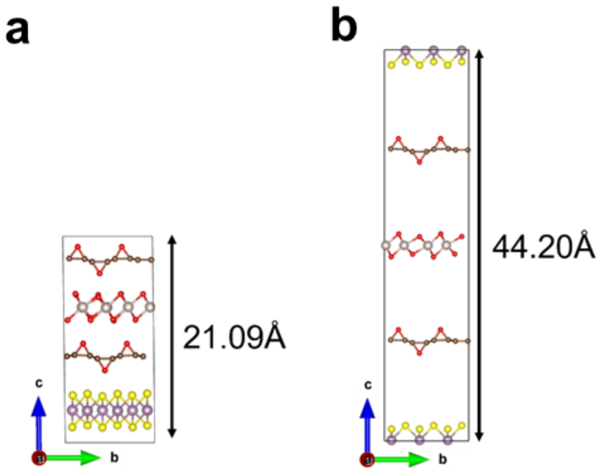


**Figure S7.** (a) DFT-optimized structure that is originally used in the manuscript, and (b) DFT-optimized structure in the simulation cell with doubled c-lattice parameter. Mo, S, C, O, and Ru are colored by yellow, purple, brown, red, and white. The length of z-axis is denoted on the right side of each structure.

**Table S3.** Comparison of the electrocatalytic hydrogen evolution performances of $MoS_2$ based nanohybrid materials in alkaline media.

| Material | Electrode | electrolyte | Overpotential (at 10 mA $cm^{-2}$) | Reference |
|---|---|---|---|---|
| **$MoS_2$/graphene** | Glassy carbon | 1M KOH | ~150 mV | [14] |
| **$Ni(OH)_2/MoS_2$** | Carbon cloth | 1 M KOH | 80 mV | [15] |
| **$MoS_2$/NiCo LDH** | Carbon fiber paper | 1M KOH | 78 mV | [16] |
| **Defect rich $MoS_2$** | Glassy carbon | 1M KOH | 111 mV | [17] |
| **$Co(OH)_2/MoS_2$** | Glassy carbon (carbon black is included in the ink) | 1M KOH | 89 mV | [18] |
| **$MoS_2$/MoP/N-doped holey carbon** | Glassy carbon | 1M KOH | 93 mV | [19] |
| **Co-$MoS_2/Mo_2CT_x$** | Glassy carbon | 1 M KOH | 112 mV | [20] |
| **Co-BDC/$MoS_2$** | Glassy carbon | 1 M KOH | 248 mV | [21] |
| **$Ni_2P/MoS_2$/N:CNT** | Glassy carbon | 1 M KOH | 152.1 mV | [22] |
| **$MoS_2/Co_9S_8/Ni_3S_2$** | Ni foam | 1 M KOH | 103 mV | [23] |
| **$MoS_2$/graphene** | Glassy carbon | 1 M KOH | 183 mV | [24] |
| **$MoS_2/NiS_x$** | Glassy carbon | 1M KOH | 100 mV | [25] |
| **$MoS_2:MoNi_4/MoO_2$** | Glassy carbon | 1M KOH | 155.6 mV | [26] |
| **$MoO_2/MoS_2$/C** | Glassy carbon | 1M KOH | 91 mV | [27] |
| **Co single atom/ $MoS_2$/TiN** | Glassy carbon | 1M KOH | 131.9 | [28] |
| **MSGR7.5** | Glassy carbon | 1M KOH | 97 mV | This work |

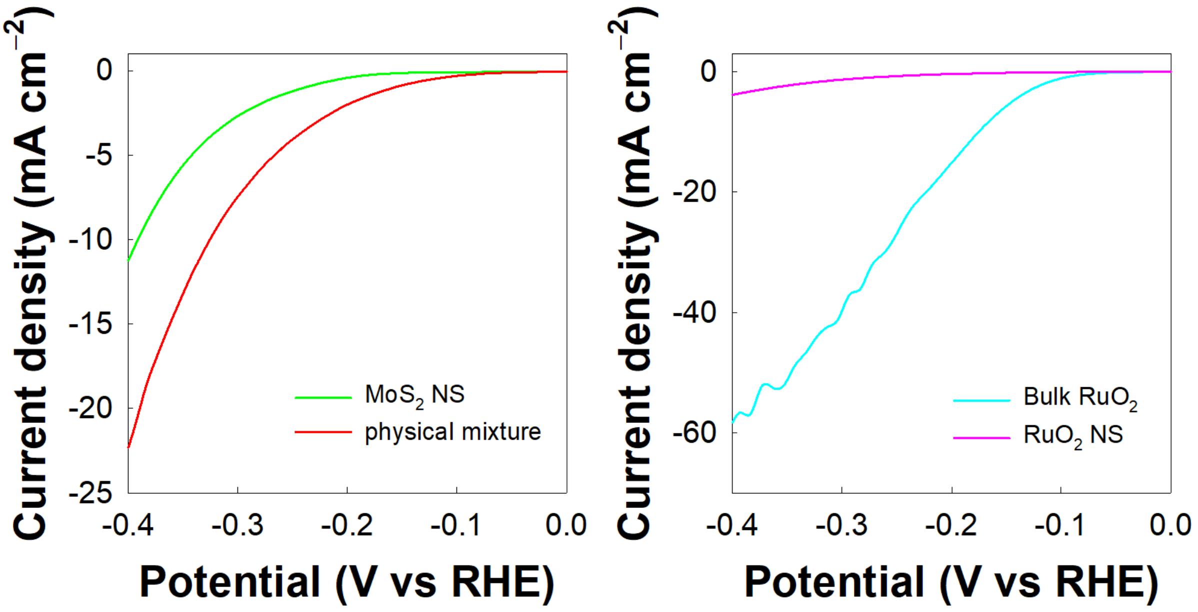


**Figure S8.** LSV curves of hydrogen evolution reaction (HER) for (left) the physical mixture of $MoS_2/RuO_2$/prGO NS and $MoS_2$ NS, and (right) bulk $RuO_2$ and $RuO_2$ NS.

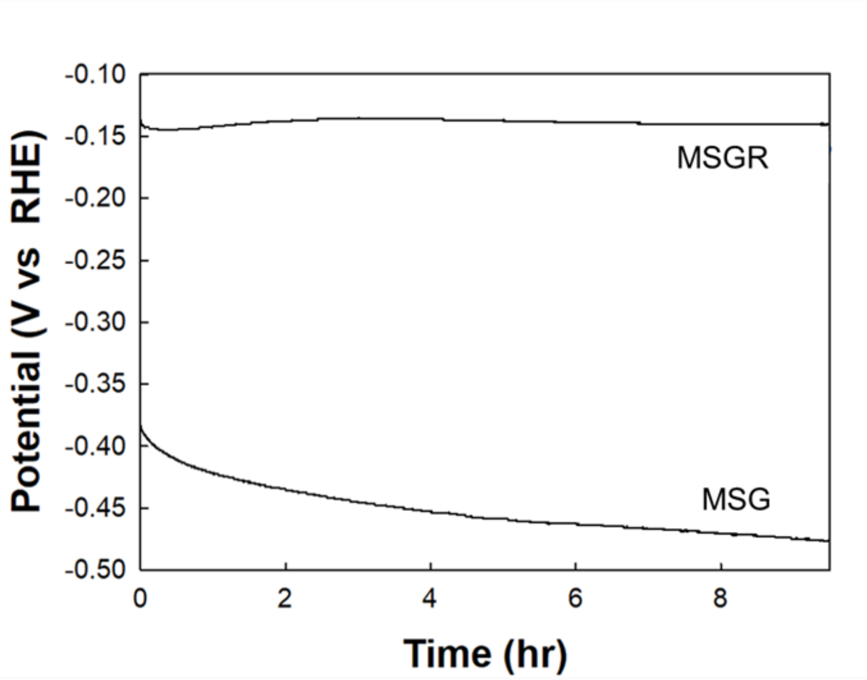


**Figure S9.** Chronopotentiometry data of **MSGR** and **MSG** nanohybrids.

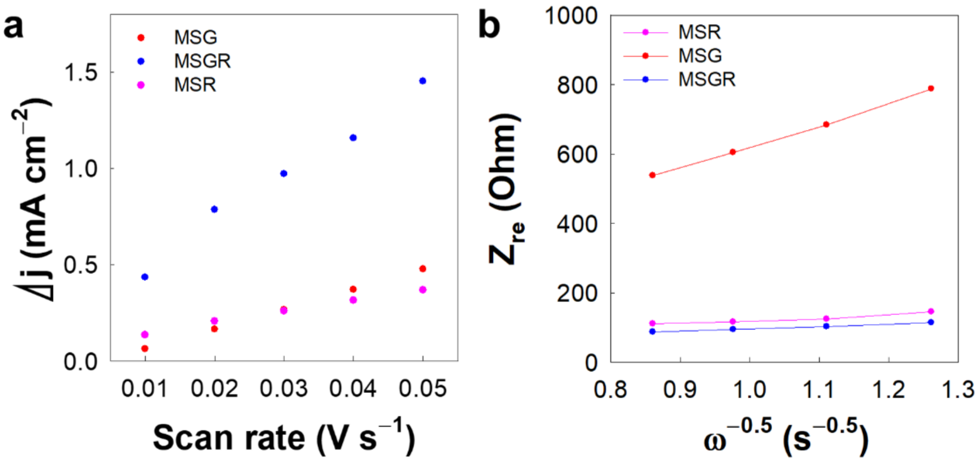


**Figure S10.** (a) Charging current difference plots and (b) plots of the real part of impedance as a function of the inverse square root of angular frequency in Warburg region for **MSGR**, **MSG**, and **MSR** nanohybrids.

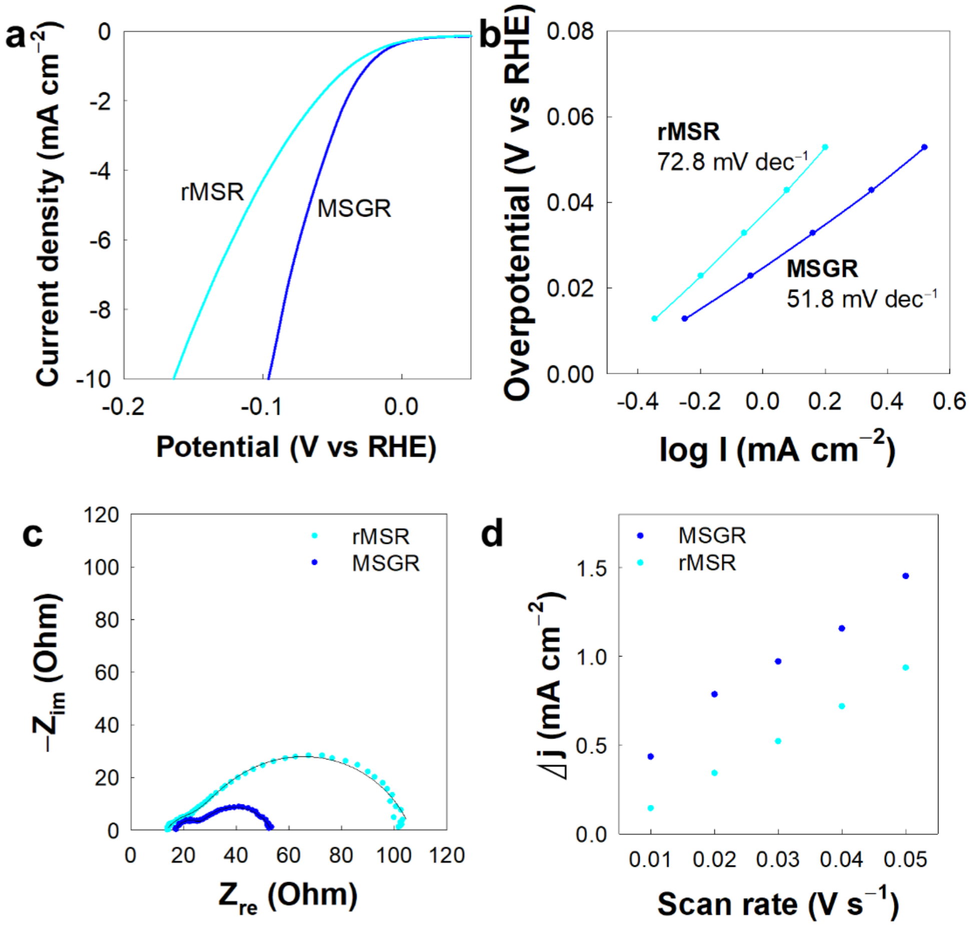


**Figure S11.** (a) LSV curves, (b) Tafel slopes, (c) EIS measurement at −0.3 V (vs. RHE), and (d) charging current differences plots of **rMSR** and **MSGR** nanohybrids.

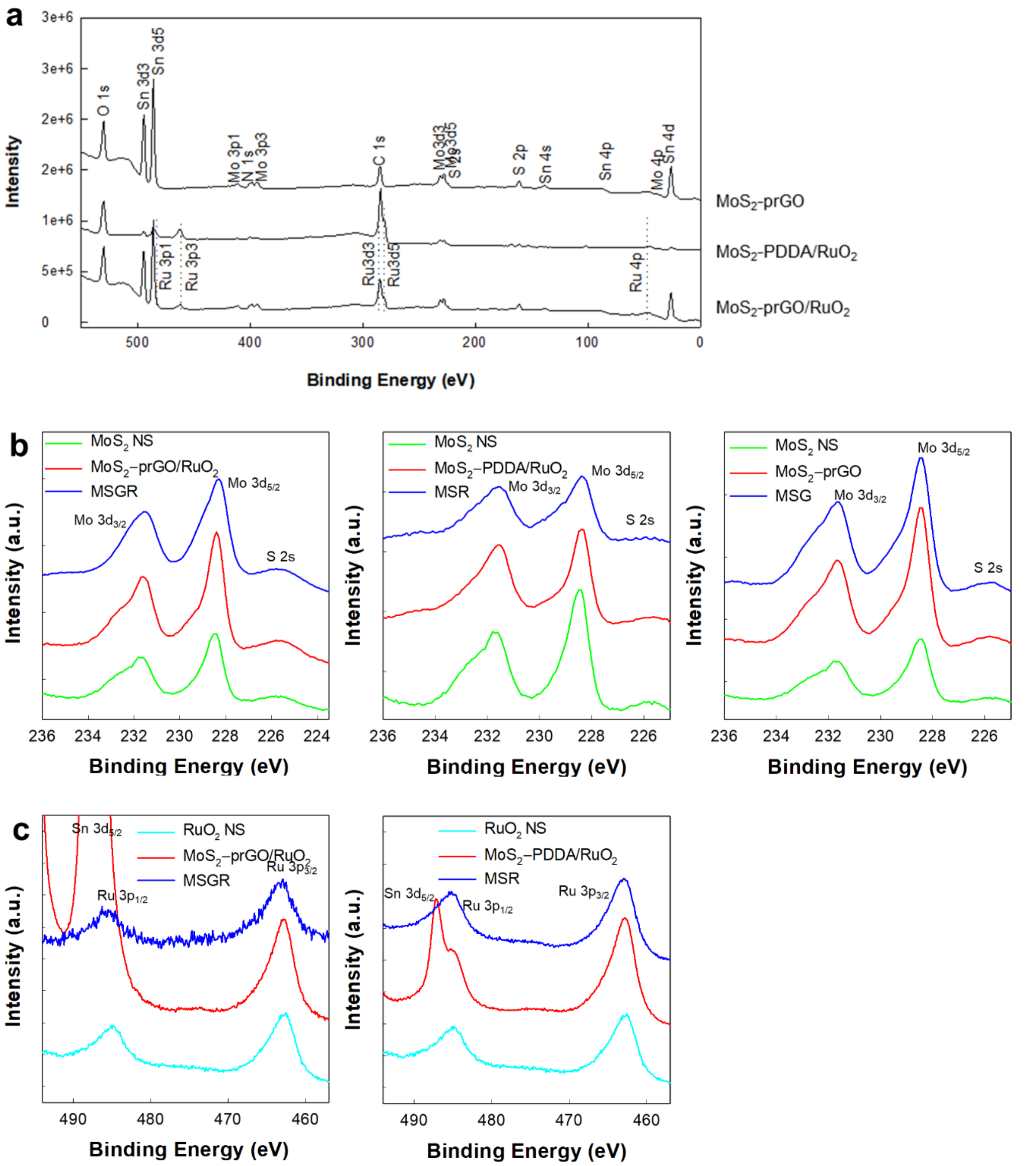


**Figure S12.** (a) Full survey XPS, (b) Mo 3d XPS, and (c) Ru 3p XPS data for the LbL films of $MoS_2$–prGO, $MoS_2$–PDDA/$RuO_2$, and $MoS_2$–prGO/$RuO_2$ NSs.

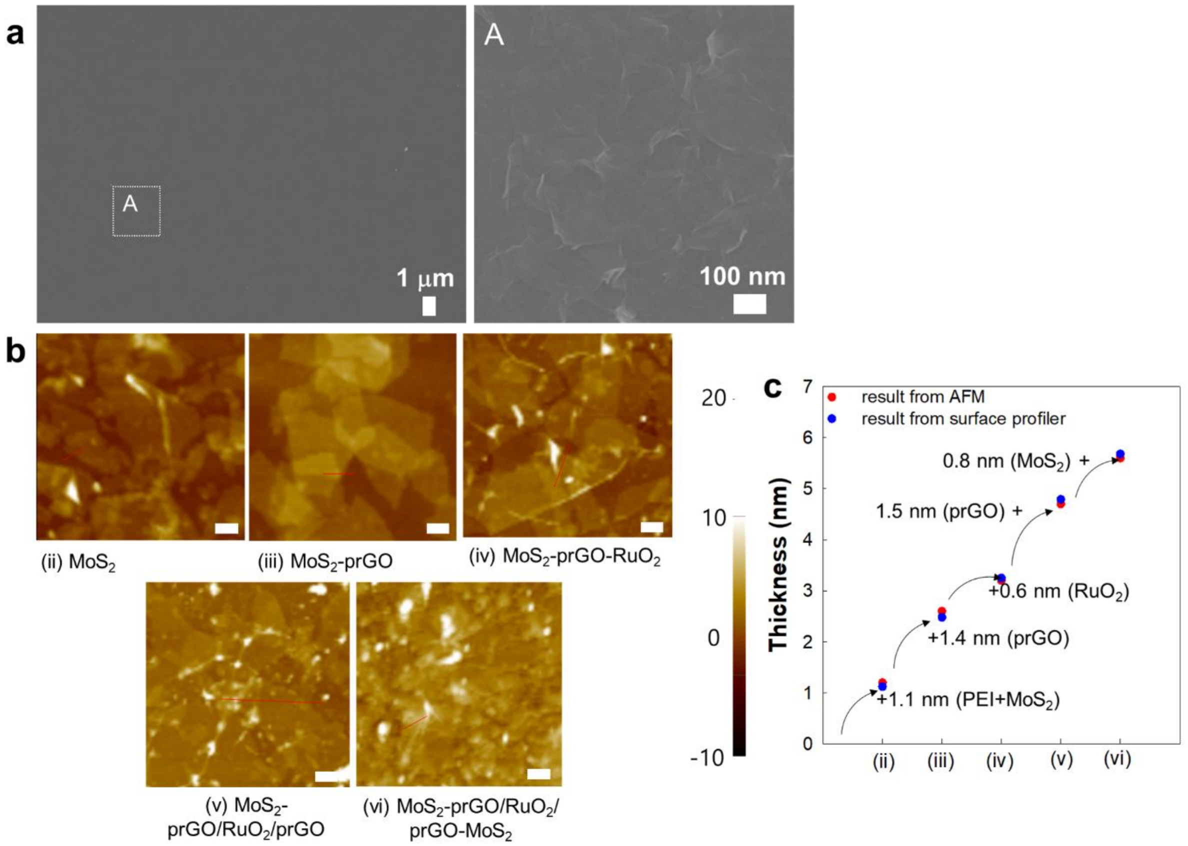


**Figure S13.** (a) Top-view FE-SEM image, (b) AFM images (the scale bar stands for 0.1 μm and red line is used to determine the height of film), and (c) thickness distribution plot of LbL film measured by AFM and surface profiler (the thickness measured by surface profiler is averaged over 3 scans).

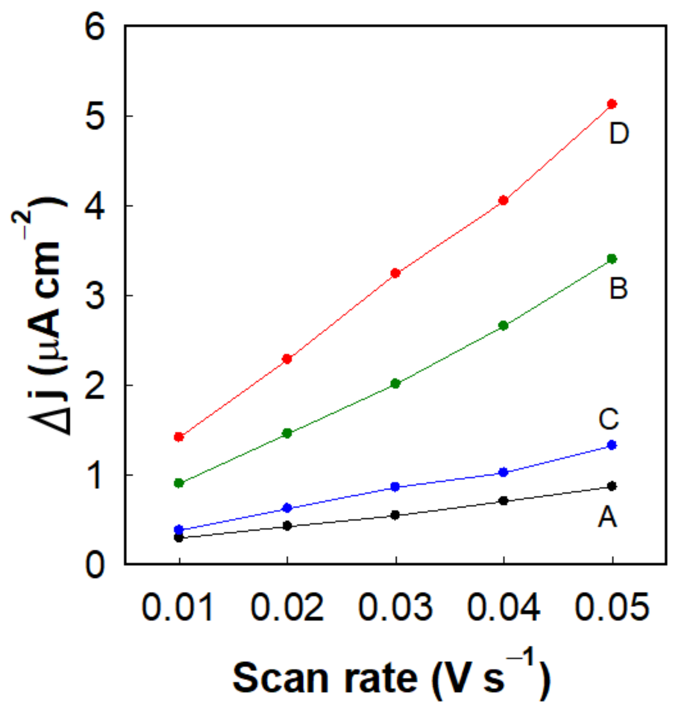


**Figure S14.** Charging current difference plots of $MoS_2$ NSs stacked with various types of conductive layers such as monolayer prGO NS (A), monolayer PDDA-$RuO_2$ NS (B), trilayer prGO/rGO/prGO NS (C), and trilayer prGO/$RuO_2$/prGO (D).

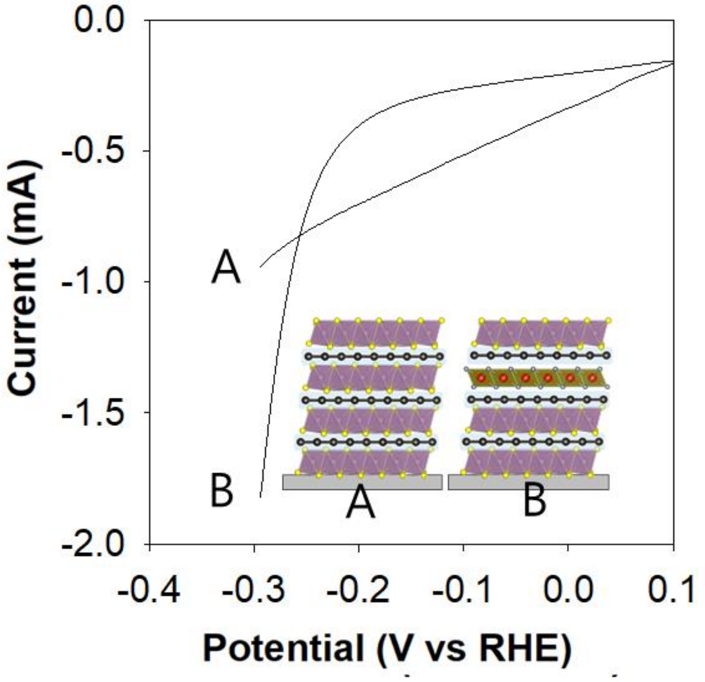


**Figure S15.** LSV curves of 4 $MoS_2$ layers without prGO/$RuO_2$/prGO layer (A) and 3 $MoS_2$ layers with 1 prGO/$RuO_2$/prGO layer (B).

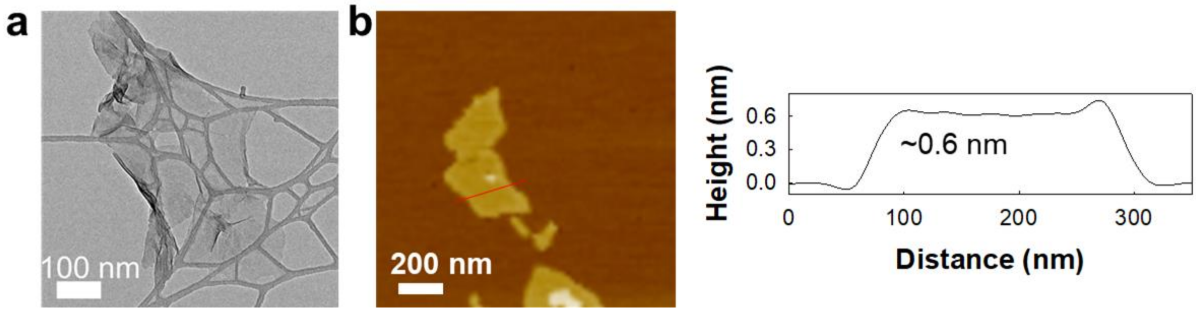


**Figure S16.** (a) TEM image and (b) AFM image of $MnO_2$ NS.

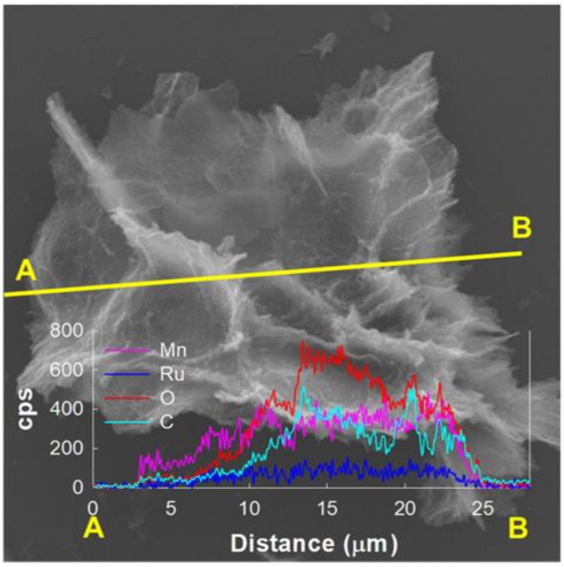


**Figure S17.** EDS–elemental map of **MOGR** nanohybrid.

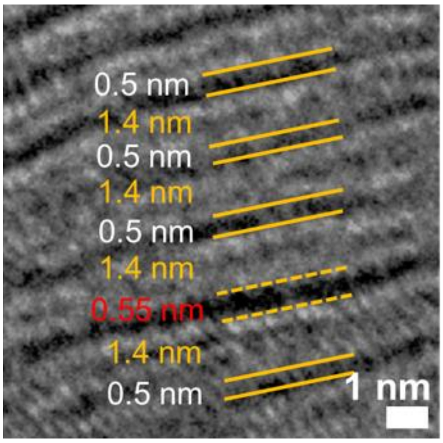


**Figure S18.** HR-TEM image of **MOGR**.

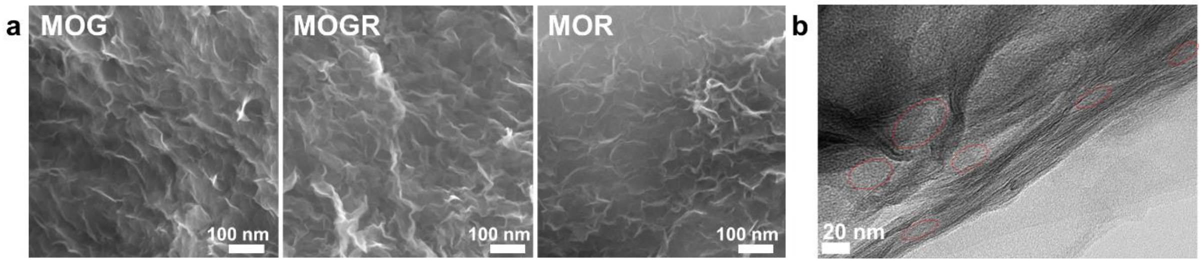


**Figure S19.** (a) FE-SEM images of **MOG**, **MOGR** and **MOR** nanohybrids. (b) HR-TEM image of **MOGR** (The red circles stand for the formation of mesopore with the size of less than 50 nm).

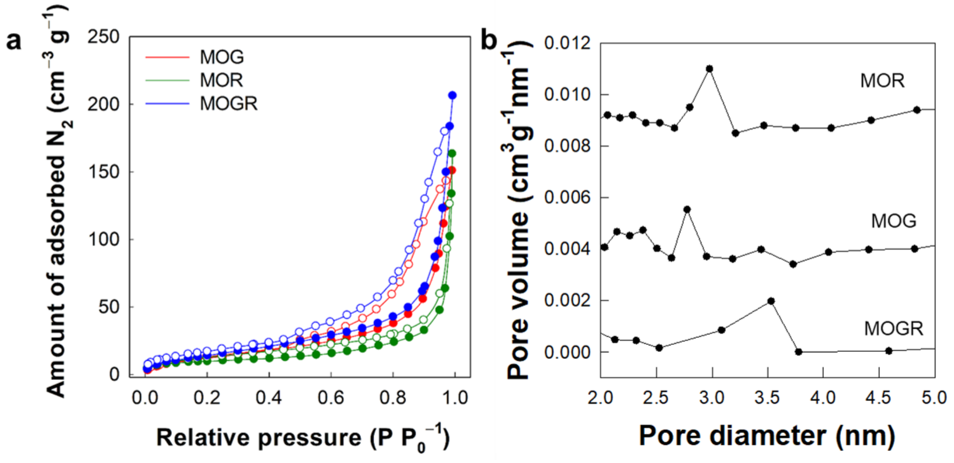


**Figure S20.** (a) $N_2$ adsorption–desorption isotherms and (b) Barrett–Joyner–Halenda (BJH) pore-size distribution plots of **MOG**, **MOR**, and **MOGR** nanohybrids.

**Table S4.** Results of non-linear least-squares curve fittings for the Mn K-edge extended X-ray absoption fine structure (EXAFS) spectra for **MOG**, **MOR**, and **MOGR** nanohybrids.

| Material | Bond pair | CN | R (Å) | $\sigma^2$ [$10^{-3} \times$ Å$^2$] |
|---|---|---|---|---|
| **MOGR** | (Mn–O) | 5.0 | 1.90 | 3.006 |
| | (Mn–Mn) | 6.0 | 2.85 | 4.035 |
| **MOR** | (Mn–O) | 4.4 | 1.90 | 4.970 |
| | (Mn–Mn) | 4.6 | 2.83 | 9.870 |
| **MOG** | (Mn–O) | 4.0 | 1.91 | 4.535 |
| | (Mn–Mn) | 4.5 | 2.84 | 5.291 |

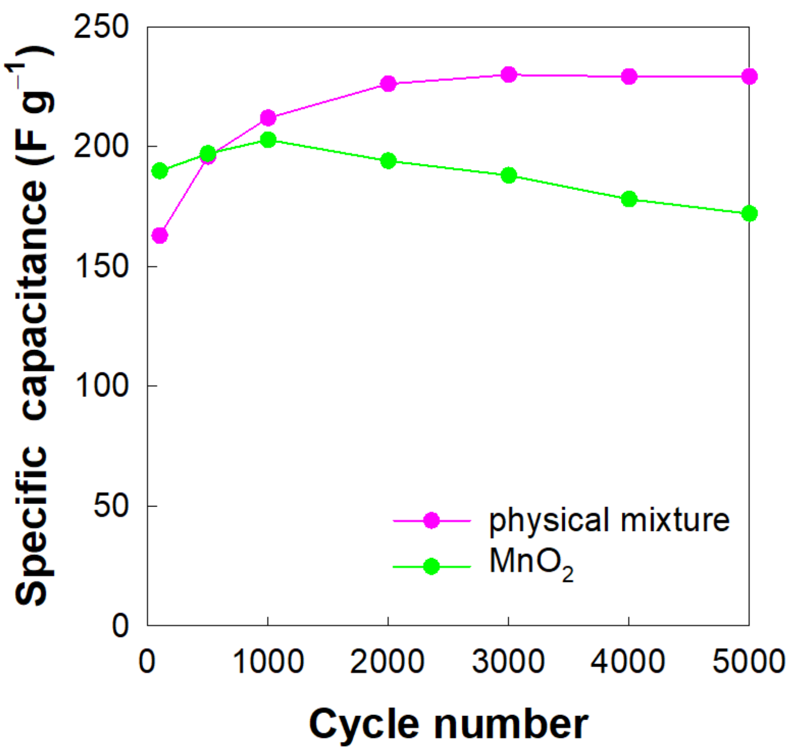


**Figure S21.** Capactiance retention plots of $MnO_2$ NS and the physical mixture of $MnO_2$, $RuO_2$, and rGO NSs.

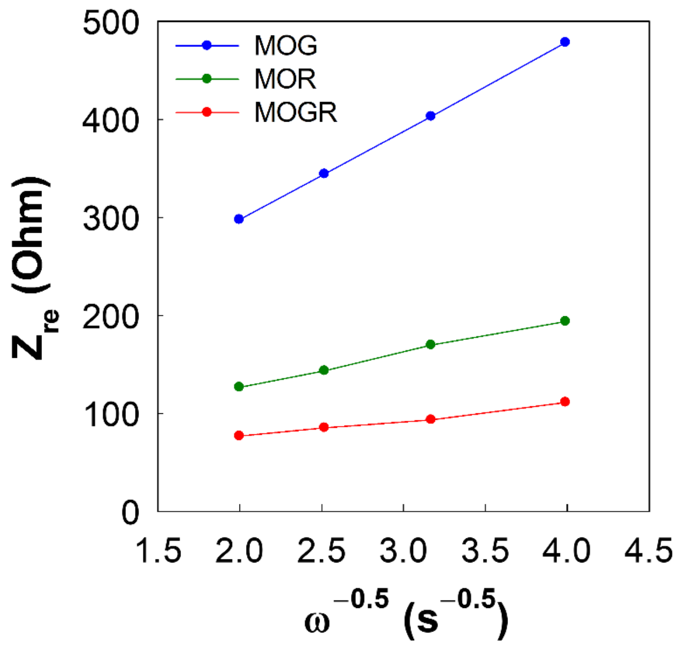


**Figure S22.** Plots of the real part of impedance as a function of the inverse square root of angular frequency in Warburg region for **MOG**, **MOR**, and **MOGR** nanohybrids.

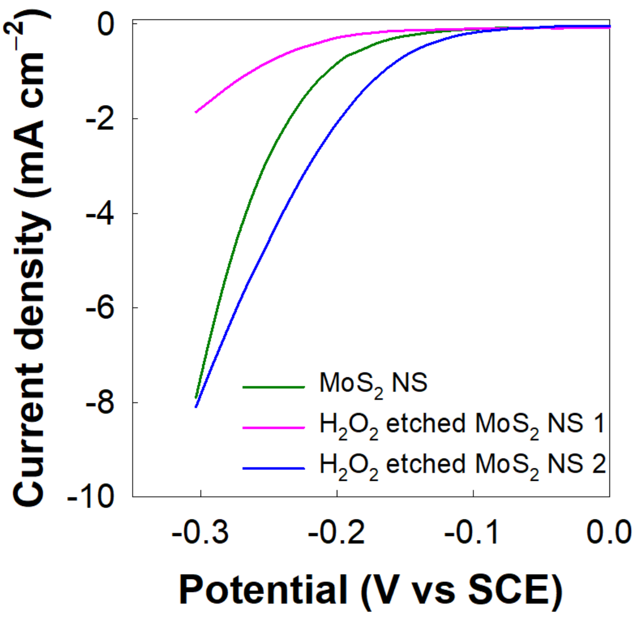


**Figure S23.** LSV curves of $MoS_2$ NSs before and after the introduction of additional sulfur vacancy with $H_2O_2$ etching.[29] For the creation of sulfur vacancy, $MoS_2$ colloid was reacted with $H_2O_2$ solution (1 mol $L^{-1}$) for 180 (1)/60 (2) sec.

References

[1] H. S. S. R. Matte, A. Gomathi, A. K. Manna, D. J. Late, R. Datta, S. K. Pati, C. N. R. Rao, *Angew. Chem. Int'l Ed.* **2010**, *49*, 4059.

[2] K. Fukuda, T. Saida, J. Sato, M. Yonezawa, Y. Takasu, W. Sugimoto, *Inorg. Chem.* **2010**, *49*, 4391.

[3] P. Xiong, R. Ma, N. Sakai, L. Nurdiwijayanto, T. Sasaki, *ACS Energy Lett.* **2018**, *3*, 997.

[4] J. Zhu, J. He, *Nanoscale* **2012**, *4*, 3558.

[5] K. Kai, Y. Yoshida, H. Kageyama, G. Saito, T. Ishigaki, Y. Furukawa, J. Kawamata, *J. Am. Chem. Soc.* **2008**, *130*, 15938.

[6] S.-J. Hwang, J.-H. Choy, *J. Phys. Chem. B* **2003**, *107*, 5791.

[7] G. Kresse, J. Furthermuller, *Phys. Rev. B* **1996**, *54*, 11169.

[8] J. P. Perdew, K. Burke, M. Ernzerhof, *Phys. Rev. Lett.* **1996**, *77*, 3865.

[9] S. Crimme, J. Antony, S. Ehrlich, H. A. Krieg, *J. Chem. Phys.* **2010**, *132*, 154104.

[10] H. J. Monkhorst, J. D. Pack, *Phys. Rev. B* **1976**, *13*, 5188.

[11] P. E. Blochl, *Phys. Rev. B* **1994**, *50*, 17953.

[12] C. C. L. McCrory, S. Jung, J. C. Peters, T. F. Jaramillo, *J. Am. Chem. Soc.* **2013**, *135*, 16977.

[13] E.-J. Oh, T. W. Kim, K. M. Lee, M.-S. Song, A.-Y. Jee, S. T. Lim, H.-W. Ha, M. Lee, J.-H. Choy, S.-J. Hwang, *ACS Nano* **2010**, *4*, 4437.

[14] H. Li, K. Yu, C. Li, Z. Tang, B. Guo, X. Lei, H. Fu, Z. Zhu, *Sci. Rep.* **2015**, *5*, 18730.

[15] B. Zhang, J. Liu, J. Wang, Y. Ruan, X. Ji, K. Xu, C. Chen, H. Wan, L. Miao, J. Jiang, *Nano Energy* **2017**, *37*, 74.

[16] J. Hu, C. Zhang, L. Jiang, H. Lin, Y. An, D. Zhou, M. K. H. Leung, S. Yang, *Joule* **2017**, *1*, 383.

[17] M. A. R. Anjum, H. Y. Jeong, M. H. Lee, H. S. Shin, J. S. Lee, *Adv. Mater.* **2018**, *30*, 1707105.

[18] Y. Luo, X. Li, X. Cai, X. Zou, F. Kang, H.-M. Chen, B. Liu, *ACS Nano* **2018**, *12*, 4565.

[19] Z. Wu, , M. Song, X. Liu, *J. Electrochem. Soc.* **2018**, *165*, F976.

[20] J. Liang, C. Ding, J. Liu, T. Chen, W. Peng, Y. Li, F. Zhang, X. Fan, *Nanoscale* **2019**, *11*, 10992.

[21] D. Zhu, J. Liu, Y. Zhao, Y. Zheng, S.-Z. Qiao, *Small* **2019**, *15*, 1805511.

[22] M. Kim, M. A. R. Anjum, M. Lee, B. J. Lee, J. S. Lee, *Adv. Funct. Mater.* **2019**, *29*, 1809151.

[23] Y. Yang, H. Yao, Z. Yu, S. M. Islam, H. M. He, Y. Yue, K. Xu, W. Hao, G. Sun, H. Li, S. Ma, P. Zapol, M. G. Kanatzidis, *J. Am. Chem. Soc.* **2019**, *141*, 10417.

[24] X. Yu, G. Zhao, S. Gong, C. Liu, C. Wu, P. Lyu, G. Maurin, N. Zhang, *ACS Appl. Mater. Interfaces* **2020**, *12*, 24777.

[25] C. Bae, T. A. Ho, H. Kim, S. Lee, S. Lim, M. Kim, H. Yoo, J. M. Montero-Moreno, J. H. Park, H. Shin, *Sci. Adv.* **2017**, *3*, e1602215.

[26] V. K. Singh, U. Gupta, B. Mukherjee, *ACS Appl. Nano Mater.* **2021**, 4, 886.

[27] F. Gong, M. Liu, S. Ye, L. Gong, G. Zeng, L. Xu, X. Zhang, Y. Zhang, L. Zhou, S. Fang, J. Liu, *Adv. Funct. Mater.* **2021**, 31, 2101715.

[28] T. L. L. Doan, D. C. Nguyen, S. Prabhakaran, D. H. Kim, D. T. Tran, N. H. Kim, J. H. Lee, *Adv. Funct. Mater.* **2021**, 31, 2100233.

[29] X. Wang, Y. Zhang, H. Si, Q. Zhang, J. Wu, L. Gao, X. Wei, Y. Sun, Q. Liao, Z. Zhang, K. Ammarah, L. Gu, Z. Kang, Y. Zhang, *J. Am. Chem. Soc.* **2020**, *142*, 4298.
